# Development of multiple input supply based modified SEPIC DC–DC converter for efficient management of DC microgrid

**DOI:** 10.1038/s41598-024-61713-z

**Published:** 2024-05-14

**Authors:** B. Nagi Reddy, Faisal Alsaif, Ch. Rami Reddy, Sunkara Sunil Kumar

**Affiliations:** 1 Department of EEE, Vignana Bharathi Institute of Technology, Hyderabad, India; 2 Department of Electrical Engineering, College of Engineering, King Saud University, 11421 Riyadh, Saudi Arabia; 3 Department EEE, Joginpally B R Engineering College, Hyderabad, India 500075; 4 Vignan’s Foundation for Science Technology and Research, Vadlamudi, India

**Keywords:** Multi-input SEPIC converter, DC micro grid, Efficient management, PV applications, Engineering, Electrical and electronic engineering

## Abstract

The development of DC microgrids is reliant on multi-input converters, which offer several advantages, including enhanced DC power generation and consumption efficiency, simplified quality, and stability. This paper describes the development of a multiple input supply based modified SEPIC DC–DC Converter for efficient management of DC microgrid that is powered by two DC sources. Here Multi-Input SEPIC converter offers both versatility in handling output voltage ranges and efficiency in power flow, even under challenging operating conditions like lower duty cycle values. These features contribute to the converter's effectiveness in managing power within a DC microgrid. In this configuration, the DC sources can supply energy to the load together or separately, depending on how the power switches operate. The detailed working states with equivalent circuit diagrams and theoretical waveforms, under steady-state conditions, are shown along with the current direction equations. This paper also demonstrates the typical analysis of large-signal, small-signal, steady-state modeling techniques and detailed design equations. The proposed configuration is validated through the conceptual examination using theoretical and comprehensive MATLAB simulation results. Detailed performance analysis has been done for different cases with various duty ratios. Finally, to show the competitiveness, the multi-input SEPIC topology is compared with similar recent converters.

## Introduction

To fulfill the demands of energy caused by the rising human population, the energy resources from renewable and non-renewable are being overused^1^. Sustainable resources cannot fulfill the demand, which take more time to form and deplete rapidly by decreasing usage^2^. The only way to extract energy is from renewable energy resources, which present in plenty of amounts and do not deplete as well^3^. The best renewable resource is solar energy, which only comes from a DC source. We need to implement it with a better power electronic converter by transferring energy to the load side where the demands can be fulfilled as well^4–6^. Compared to AC energy, DC has more advantages for more applications, and its usage has increased^7^. The best power electronic converter to transfer energy from source to load is the single-ended primary inductor converter (SEPIC), which has many advantages and is considered the best converter compared to traditional converters^8^.

The primary aspect of implementing SEPIC converter is the we need to have a storage system as a second source in the SEPIC converter, the best way to store DC energy is by Battery over fuel and other different storage systems. Electric batteries are becoming increasingly important for storing energy from renewable sources, such as solar and wind power^9^. Renewable energy sources are intermittent, meaning they do not always produce electricity^10^. Electric batteries can also be used to provide backup power in case of a power outage. The stored or generated energy can be given to a separate load or to a DC micro grid where it can be further supplied to different stations as well^11^. Integrating to a DC micro grid can be implemented in a usage able way, where the generated can be in a bulk amount and supplied in a bulk amount as well. DC microgrids present numerous advantages upon close examination and comparison with AC microgrids^12^. Firstly, they offer higher efficiency and lower losses by eliminating the need for multiple converters in a system. Second, they enable the smooth integration of diverse DC power sources, such as energy storage systems, DC source 1 and DC source 2, into a unified DC bus. This simplifies the overall system design and interface requirements^13^. Thirdly, they enhance the efficiency of supplying power for various devices, from electric vehicles on the road to the LED lights illuminating our homes and businesses^14^. One of the key benefits of DC microgrids is that they do away with the need to synchronize generators. This allows them to operate at their most efficient speeds, maximizing power output.

Additionally, DC systems eliminate the complexity of synchronizing buses when connecting multiple microgrids. These advantages and the growing prevalence of DC-powered devices like computers, laptops, LED lights, and data centres make DC microgrids a compelling solution for future energy demands^15^. As AC systems may not be readily available in all locations, DC microgrids offer a versatile and adaptable alternative.

A SEPIC converter is a versatile DC–DC configuration that can adjust its output voltage to be higher, lower, or even equal to the input voltage. This control is achieved by electronically adjusting a switch within the circuit. The SEPIC's design is essentially a boost configuration combined with a buck-boost configuration operating in reverse^16–20^. This unique combination gives SEPIC converters a key advantage over traditional buck-boost converters: The input and output voltages are kept in phase. In addition, the SEPIC responds to a short-circuit output with greater grace since it uses a series capacitor to move energy from the input to the output^21–28^. Another advantage of SEPIC converters is their ability to shut down completely. When the switch is turned off entirely, the output voltage drops to zero, although a temporary surge of energy may be released during this process^29–32^. This complete shutdown capability makes SEPICs well-suited for situations where battery voltage fluctuates significantly^33–38^. For example, a lithium-ion battery's voltage typically drops from 4.2 to 3 V as it discharges. If a device requires a steady 3.3 V, a SEPIC converter can efficiently maintain that voltage level even with a fluctuating battery supply.

But building on the research, this paper introduces a novel of DC–DC SEPIC converter where it is suitable for DC Power micro grids applications^39,40^. It can also be used for DC standalone applications where it can be reliable. As mentioned earlier, the DC energy is more advantageous than AC energy compared to the daily and important power applications^41–45^. The reason to introduce the SEPIC converter among the traditional converter is that it produces a wide range of input and output at the lower value of duty cycles, here electrical stress across electrical switches is less and the power loss is also less by, which there is an increase in efficiency (above 95%).

The main contribution of this paper is as follows.

- Introduction of a Highly efficient SEPIC (Single-Ended Primary Inductor Converter) configuration for power management in DC microgrids.
- Performance evaluation based on the (R–H) criteria, considering both small signal and large signal modeling to account for linear and non-linear devices.
- Through assessment of Multi-Input SEPIC configuration efficiency through simulation results.
- Detailed analysis of waveforms associated with switches and energy storing elements in the system.
- Contribution enhances understanding of system behavior, stability, and efficiency, providing valuable insights for real-world applications.

The paper is meticulously organized as follows. Section “Literature survey” provides an insightful overview of various converters integrated with the Single-Ended Primary Inductor Configuration (SEPIC), setting the stage for the proposed system. Section “Methodology” elucidates the different modes of operation within the SEPIC configuration, deriving them through state space modeling. Moving to Section “Analysis & design”, a comprehensive analysis of SEPIC is presented, incorporating average large signal modelling, small signal modeling, (R–H) criteria assessment, steady-state modelling, and the design intricacies of key components like capacitors and inductors. This section also encompasses efficiency and voltage stress calculations. Section “Results” details the results obtained from simulations conducted on diverse circuits, evaluating the proposed converter's performance across different cases. Furthermore, it discusses the comparative analysis of the proposed topology with existing configurations. Section “Conclusion” succinctly concludes the paper, summarizing key findings and contributions, and providing closure to the proposed configuration's exploration.

## Literature survey

At present, the energy that can be generated by renewable is more when compared non- non-renewable energy resources in terms of the fuel that may be extinguished in the future^18^. The most efficient way to store energy is to store it in a battery using a solar PV array system^22^. Other DC storage systems cannot be used in these circumstances, where they are more disadvantages than DC batteries. The foremost disadvantage of using a fuel cell is the presence of hydrogen, the cost of hydrogen element is higher when compared to the total cost taken to a battery manufacture. Now, to supply DC voltage, which is stored by solar PV system we need an efficient DC–DC converter, which can perform the Boost and Buck operation as well, the further the DC voltage can be given DC microgrid application. From this paper, there are many DC–DC converters in the Power Electronics Concept, like Buck, Boost, Buck-Boost, Cuk, Fly-back, SEPIC and Zeta converters, etc. At our foremost need the input to the converter should be a DC supply and a DC power Storage system^23^. These terms and conditions can only be satisfied by the SEPIC converter. Where when compared to other converters will not fulfill the demands and there will more losses for different converters if won't choose SEPIC (Single Ended Primary Inductor Converter) and efficiency will a significant factor in determining such a converter.

Now, when Boost converter is compared with the SEPIC the output voltage can only be increased and the output cannot be bucked further as the paper states that the output voltage can be given to DC power micro grid application, where the applications can be of less voltage and more voltages, Where Buck and Boost of output voltage is also required. Where in SEPIC converter, the Buck and Boost of the output voltage can be done^24^. The boost converter cannot be used as energy storage system as well. Now when Buck-boost converter is used here the output voltage can be increased or decreased as well but the main disadvantage is that the input voltage should be given with a specific limit and the circuit complexity would be increased as well. There no inductor at the primary where the current cannot be in continuous mode, here also the storage system can be implemented further^25^. The Buck-Boost converters main drawback is that its larger and heavier than traditional one. In Flyback converter, the input can also be varied, but the drawback is that its cost is more. In terms of the construction also the two different converters topologies, the flyback converter and the boost converter by which the additional components and wiring^26^.

In order to decrease those, dis-advantages we can implement the SEPIC converter where the cost can be less when compared to flyback converter. The 5th order converter is ZETA converter where the input can be varied and the output voltage as well. By implementing this converter instead of the SEPIC, the output side capacitor by which the output ripples can be removed, even though the input side capacitor is not present, where the current will not be continuous^31^. But the continuous current is required based on the applications of DC power microgrids. This drawback can be rectified by SEPIC converter only the remaining converter cannot be used where the inductor is not at the input side. When the other converter is to be compared with the SEPIC converter, this comes first in terms of the DC power micro grids applications, where the remaining converter's drawback can be rectified as well. To overcome by the individual drawbacks, the we can implement the combination as well^24^.

In co-ordination with the buck-boost and Cuk where the converters have same topologies of working where in combined, they can work in more efficient way. The output can be of same when compared SEPIC and the combination of Buck-Boost & Cuk converter^25^. However, the issue is the increase of complexity, where the complexity can be reduced and the desired output can be acquired as well. Further the SEPIC topology technique can be implemented by Buck and Buck-Boost converter as well, where the multiple DC voltages can be connected to the DC micro grid bus. But the main intention is not regarding the input DC but the output desired voltage, which can be supplied further to plenty of DC power applications^33^.

But when we try to implement it, the cost will be more expensive compared to implementing single SEPIC alone. Not to mention that the complexity will also be increased, which will decrease usage. Now, in urban days, people try to use more energy at day time and low energy at nighttime, to fulfil it we must have control of input and output as well. Now this can be achieved by hybrid DC–DC converter based on buck-boost and ZETA converters for DC microgrids with grid connection^31^. But the drawback is that its efficiency is less when compared to the traditional DC–DC converter. As efficiency is the main parameter to determine its performances.

## Methodology

**Figure 1:**
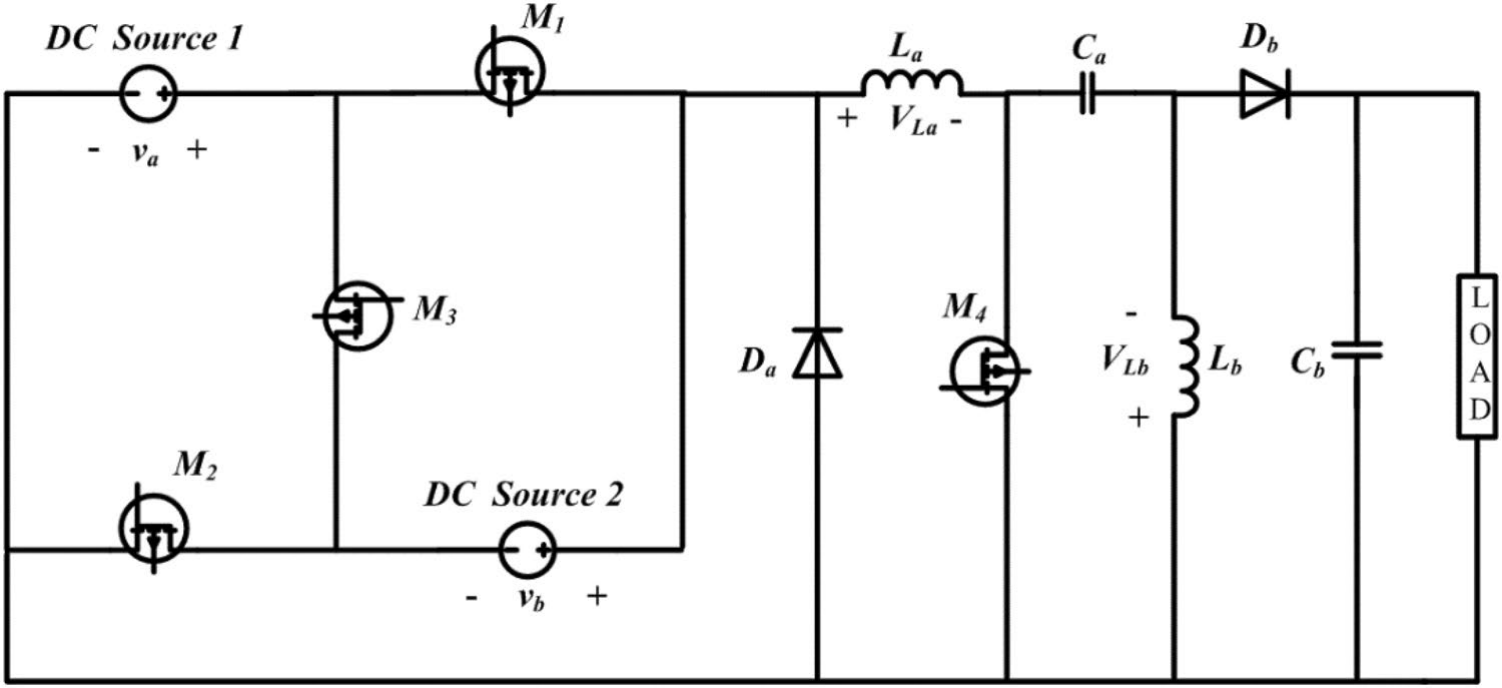
Multi-Input DC–DC configuration using SEPIC topology.

**Figure 2:**
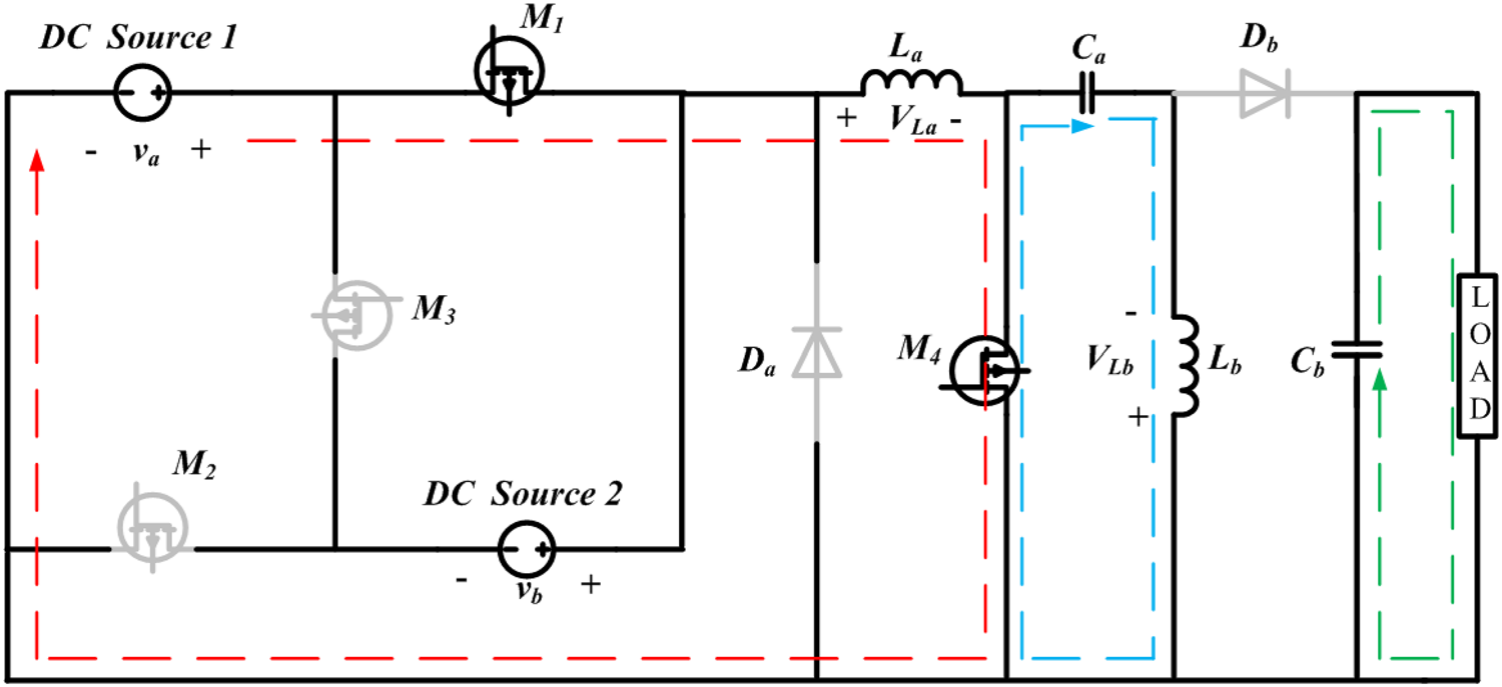
Operation of state-1 of the proposed converter.

**Figure 3:**
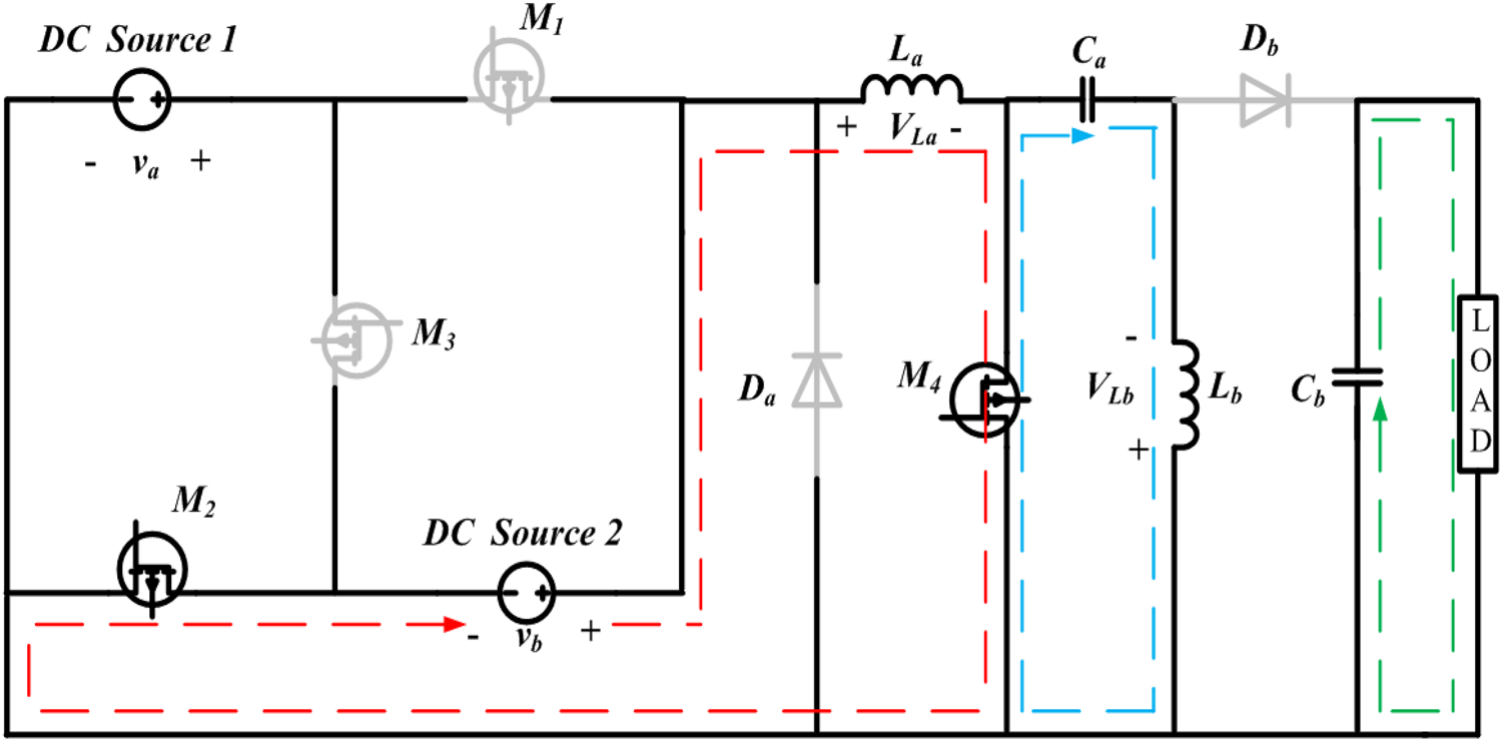
Operation of state-2 of the proposed configuration.

**Figure 4:**
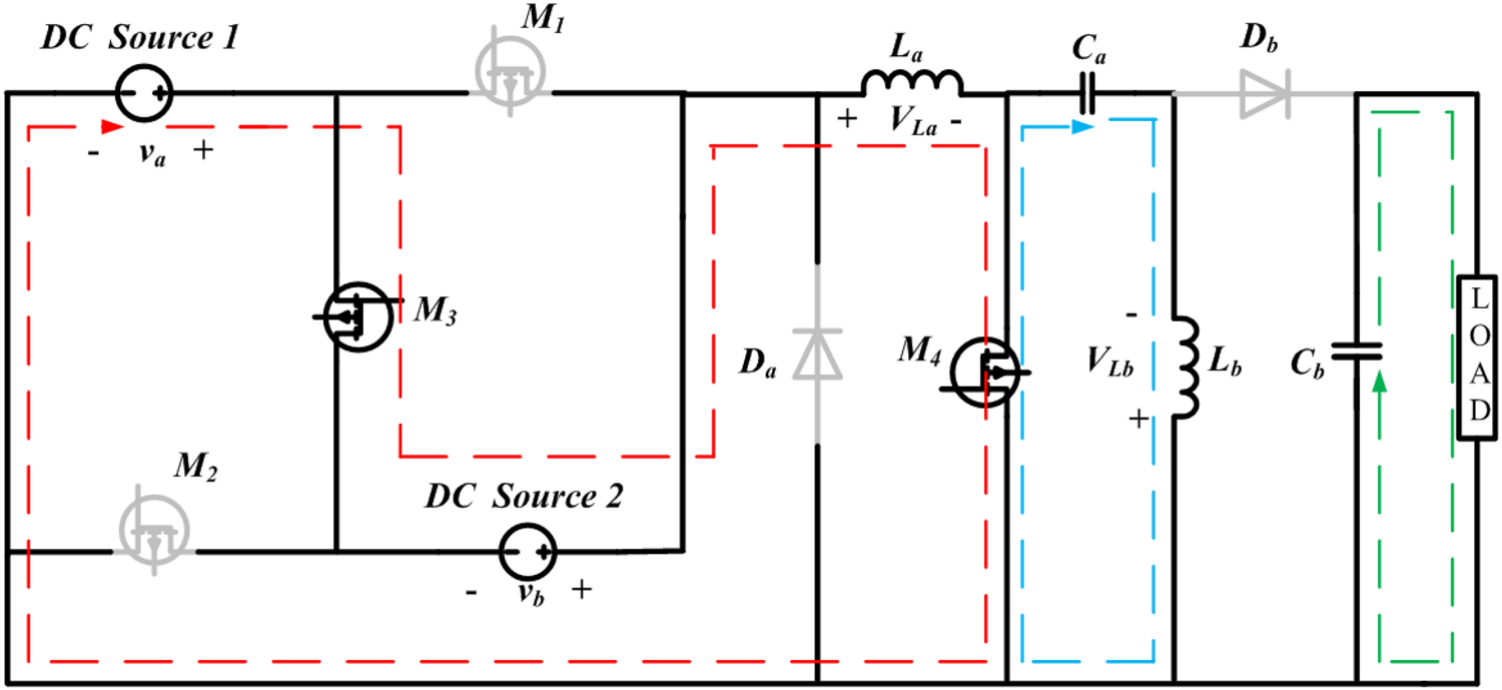
Operation of state-3 of the proposed converter.

**Figure 5:**
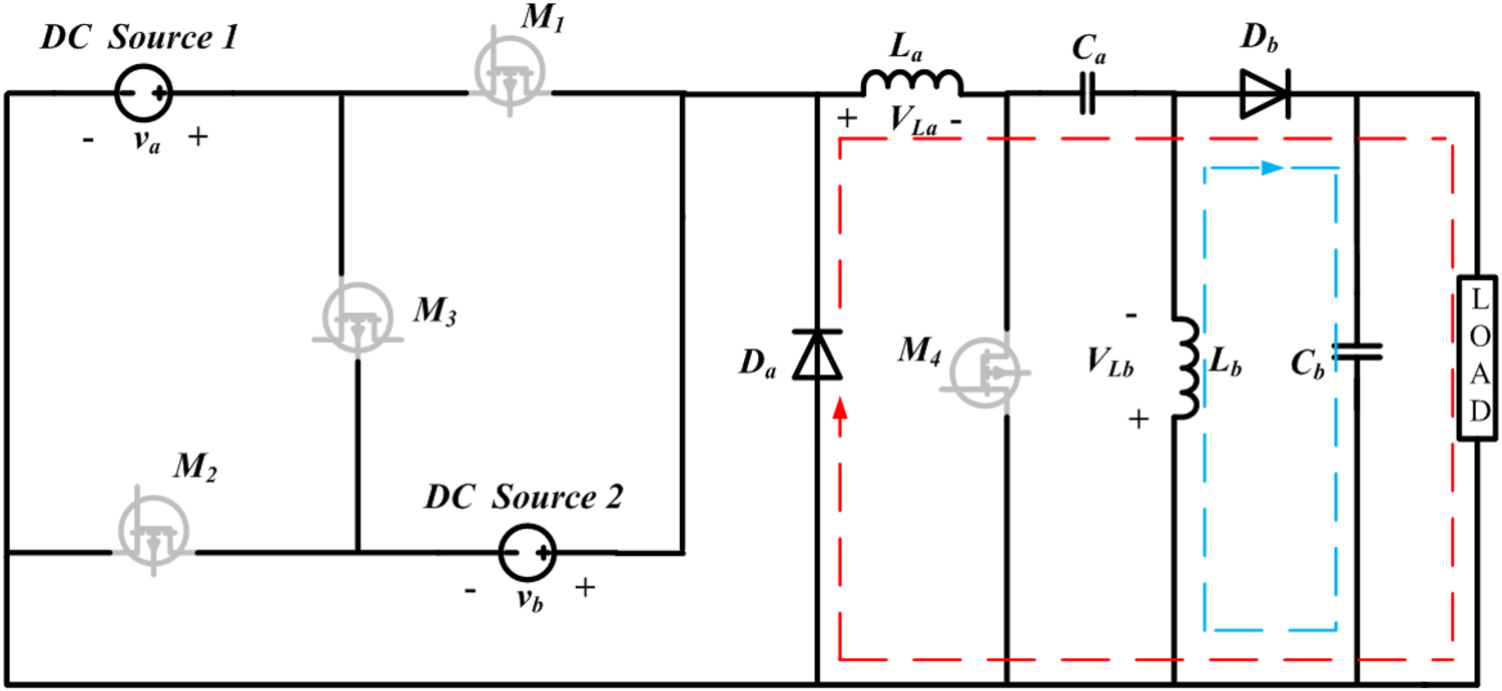
Operation of state-4 of the proposed configuration.

The traditional single-input DC–DC configuration and the newly proposed converter function similarly in terms of their basic operating principles. In each mode of operation, both configurations involve the inductor $(L)$ and capacitor $(C)$ components storing energy for a specific duration and then releasing that energy to the load. The proposed converter, depicted in Fig. 1, operates in four different states (stages 1–4), each corresponding to an equivalent network (Figs. 2, 3, 4, 5). The ideal waveforms for the suggested DC source 1 and 2 under Continuous Conduction Mode (CCM) are illustrated in Fig. 1. In Fig. 1, ${v}_{a}$ and ${v}_{b}$ represent the DC source 1 and 2's large-signal terminal voltages, respectively. ${M}_{1}$ to ${M}_{4}$ are the four MOSFET switches, Diode $a$ and Diode $b$ are two diodes, ${L}_{a}$ and ${L}_{b}$ are the inductors, ${C}_{a}$ and ${C}_{b}$ are the capacitors, and $R$ is the load resistance.

${V}_{La}$ and ${V}_{Lb}$ indicate the large-signal voltages across ${L}_{a}$ and ${L}_{b}$, respectively, while ${I}_{La}$ and ${I}_{Lb}$ represent the large-signal currents flowing through ${L}_{a}$ and ${L}_{b}$. Similarly, ${I}_{Ca}$ and ${I}_{Cb}$ denote the large-signal currents through ${C}_{a}$ and ${C}_{b}$, and ${V}_{0}$ is the large-signal voltage across *R*. The gating pulses for switches ${M}_{1}$ to ${M}_{4}$ are $VG{M}_{1}$ to ${VGM}_{4}$, respectively. ${V}_{Ca}$ and ${V}_{Cb}$ are the large-signal voltages across ${C}_{a}$ and ${C}_{b}$, and the large-signal current flowing through *R* is ${I}_{0}$.${V}_{0}$ is the steady-state voltage across *R*, and ${I}_{0}$ = ${V}_{0}$*/R* is the corresponding steady-state current. It's important to note that while the designed converter can operate with various input combinations, it is a theoretical model and does not address the practical challenges associated with integrating DC source 1 and DC source 2 systems into the grid. The different states of the Proposed converter, where the Table 1 gives the information regarding the elements which would charge or discharge, current direction which source acting as main input to the converter.

**Table 1:** Different states of the proposed configuration.

States	Source	Switches on	Switches off	Charging	Discharging	Current flow
STATE-1	DC source 1 **(**${V}_{a}$**)**	${M}_{1}, {M}_{4}$	${M}_{2}, {M}_{3}, {D}_{a},{D}_{b}$	${L}_{a}$**,** ${L}_{b}$	${C}_{a}$**,**${C}_{b}$	LOOP 1—${V}_{a }^{+}\to {M}_{1}\to {L}_{a}\to {M}_{4}\to {V}_{a }^{-}$ LOOP 2—${c}_{b}^{+}\to {L}_{b}\to {M}_{4}\to {c}_{b }^{-}$ LOOP 3—${c}_{b}^{+}\to {V}_{0}\to {c}_{b }^{-}$
STATE -2	DC source 2 (${V}_{b}$)	${M}_{2}, {M}_{4}$	${M}_{1},{M}_{3}$, ${D}_{a}$, ${D}_{b}$	${L}_{a}$**,**${L}_{b}$	${C}_{a}$**,** ${C}_{b}$	LOOP 1—${V}_{b}^{+}\to {L}_{a}\to {M}_{4}\to {M}_{2}\to {V}_{b }^{-}$ LOOP 2—${c}_{b}^{+}\to {L}_{b}\to {M}_{4}\to {c}_{b }^{-}$ LOOP 3—${c}_{b}^{+}\to {V}_{0}\to {c}_{b }^{-}$
STATE -3	DC source 1 and DC source 2 ($${V}_{a}$$ + $${V}_{b}$$)	$${M}_{3},{M}_{4}$$	$${M}_{1}$$, $${M}_{2}$$, $${D}_{a}$$,$$ {D}_{b}$$	$${L}_{a}$$**,** $${L}_{b}$$	$${C}_{a}$$**,** $${C}_{b}$$	LOOP 1—$${V}_{a }^{+}\to {M}_{3}\to {V}_{b}\to {L}_{a}\to {M}_{4}\to {V}_{a }^{-}$$ LOOP 2—$${c}_{b}^{+}\to {L}_{b}\to {M}_{4}\to {c}_{b }^{-}$$ LOOP 3—$${c}_{b}^{+}\to {V}_{0}\to {c}_{b }^{-}$$
STATE -4	No source active	${D}_{a}$**,** ${D}_{b}$	${M}_{1},{M}_{2},{M}_{3}, {M}_{4}$	${C}_{a}$, ${C}_{b}$	${L}_{a}$**,** ${L}_{b}$	LOOP 1—${L}_{a }^{+}\to {C}_{a}\to {D}_{b}\to {V}_{0}\to {D}_{a}\to {L}_{a }^{-}$ LOOP 2—${L}_{b}^{-}\to {D}_{b}\to {C}_{b}\to {L}_{b }^{+}$

### States of operations

The suggested converter's precise functioning, the corresponding state-space model, and dynamic equations in each operating state are discussed in Sections “Introduction” to Analysis & design.

#### State-1

**Figure 6:**
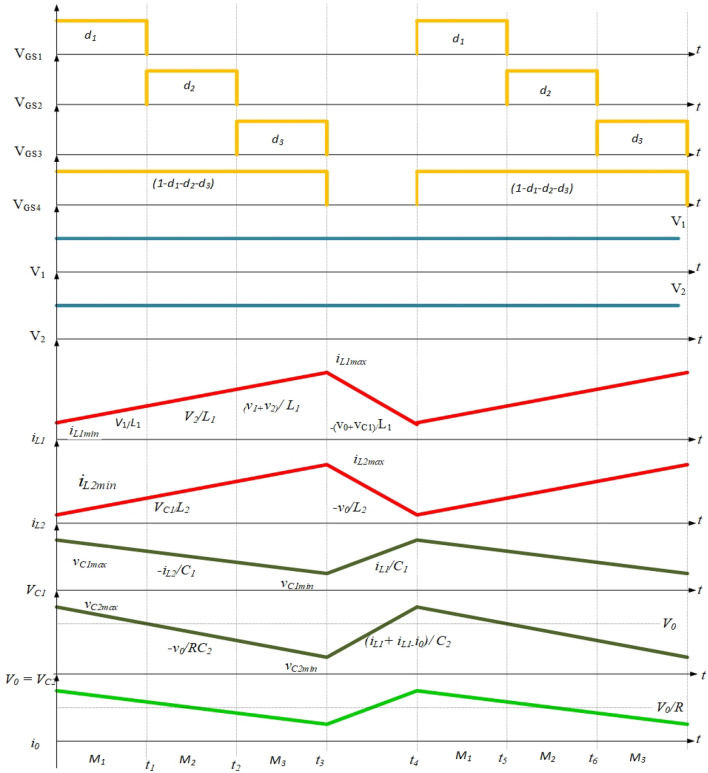
Theoretical waveforms of the proposed converter.

In State-1, the switches ${M}_{1}$ and ${M}_{4}$ are in the ON condition and ${M}_{2}$, ${M}_{3}$ Diode *a* and Diode *b* are inactive. During the operating phase from $0$ to ${t}_{1}$, equivalent to $\left({\delta }_{1 }\right)t$, as illustrated in Fig. 6, the converter exhibits specific characteristics. The equivalent circuit for this particular state of operation is presented in Fig. 2. In this interval, the inductors ${L}_{a}$ and ${L}_{b}$ undergo a charging process, and the inductor currents ${i}_{La}$ and ${i}_{Lb}$ have slopes that are determined by $\frac{{v}_{a}}{La}$ and $\frac{{v}_{Ca}}{Lb}$, respectively. The inductor current ${i}_{La}$ and ${i}_{Lb} \mathrm{their maximum values }{i}_{La}$ max and ${i}_{Lb}$ max. The energy stored in the capacitor $({C}_{b})$ is discharging along load resistance by having the slope of the voltage ${v}_{Cb}$ is $\frac{-{v}_{o}}{RCb}$. The capacitors ${C}_{a}$ starts discharging and helps ${L}_{b}$, the slopes of the capacitor voltages ${v}_{Ca}$ is $\frac{-{i}_{Lb}}{Ca}$.

Applying KVL and KCL to the circuit shown in Fig. 2, the equations presented in (1) to (5) can be derived.

1 $${v}_{La}={v}_{a}$$

2 $${v}_{Lb}={v}_{ca}$$

3 $${i}_{Ca}=-{i}_{Lb}$$

4 $${i}_{Cb}=-{i}_{0}$$

5 $${v}_{0}={v}_{cb}=-{i}_{0}R=-{i}_{Cb}R$$

The dynamic equations of State-1 is given below:

6 $$\left\{\begin{array}{c}\frac{{di}_{La}}{dt}=\frac{{v}_{a}}{La} \left({\delta }_{1}t\right)\\ \frac{{di}_{Lb}}{dt}= \frac{{v}_{{C}_{a}}}{Lb}{(\delta }_{1}t)\\ \begin{array}{c}\frac{{dv}_{{C}_{a}}}{dt}=\frac{{i}_{{L}_{b}}}{{C}_{a}}({\delta }_{1}t)\\ \frac{{dv}_{{C}_{b}}}{dt}=\frac{{v}_{o}}{{RC}_{b}}({\delta }_{1}t)\end{array}\end{array}\right.$$

The suggested system exhibits a fourth-order configuration, and the state-space model can be formulated by employing the dynamic equations corresponding to State-1 are shown in Eqs. (7) and (8). with the inductor currents. ${i}_{La}$ and ${i}_{Lb}$ and capacitor voltages ${v}_{Ca}$ and ${v}_{Cb}$ as the state variables.

7 $$\left[\begin{array}{c}\frac{{di}_{La}}{dt}\\ \frac{{di}_{Lb}}{dt}\\ \frac{{dv}_{Ca}}{dt}\\ \frac{{dv}_{Cb}}{dt}\end{array}\right]=\left[\begin{array}{cccc}0& 0& 0& 0\\ 0& 0& \frac{1}{{L}_{b}}& 0\\ 0& -\frac{1}{{C}_{a}}& 0& 0\\ 0& 0& 0& -\frac{1}{{RC}_{b}}\end{array}\right]\left[\begin{array}{c}{i}_{La}\\ {i}_{Lb}\\ {v}_{Ca}\\ {v}_{Cb}\end{array}\right]+\left[\begin{array}{cc}0& \frac{1}{{L}_{a}}\\ 0& 0\\ 0& 0\\ 0& 0\end{array}\right]\left[\begin{array}{c}{v}_{a}\\ {v}_{b}\end{array}\right]$$

8 $${v}_{o }=\left[\begin{array}{cccc}0& 0& 0& 1\end{array}\right] \left[\begin{array}{c}{i}_{La}\\ {i}_{Lb}\\ {v}_{Ca}\\ {v}_{Cb}\end{array}\right]+0$$

#### State-2

In State–2, the switches ${M}_{2}$ and ${M}_{4}$ are in the ON condition and ${M}_{1}$, ${M}_{3}$ Diode a and Diode b are inactive. During the operating phase from ${t}_{1}$ to ${t}_{2}$, which is equivalent to $\left({\delta }_{2 }\right)t$, as illustrated in Fig. 6. the converter exhibits specific characteristics. The equivalent circuit for this particular state of operation is presented in Fig. 3. In this interval, the inductors ${L}_{a}$ and ${L}_{b}$ undergo a charging process, and the inductor currents ${i}_{La}$ and ${i}_{Lb}$ have slopes that are determined by $\frac{{v}_{b}}{La}$ and $\frac{{v}_{Ca}}{Lb}$, respectively. The inductor current ${i}_{La}$ and ${i}_{Lb} \mathrm{their maximum values }{i}_{La}$ max and ${i}_{Lb}$ max. The energy stored in the capacitor $({C}_{b})$ is discharging along load resistance by having the slope of the voltage ${v}_{Cb}$ is $\frac{-{v}_{o}}{RCb}$. The capacitors ${C}_{a}$ starts discharging and helps ${L}_{b}$, the slopes of the capacitor voltages ${v}_{Ca}$ is $\frac{-{i}_{Lb}}{Ca}$.

Applying KVL and KCL to the circuit shown in Fig. 3, the equations presented in (9) to (13) can be derived.

9 $${v}_{La}={v}_{b}$$

10 $${v}_{Lb}={v}_{ca}$$

11 $${i}_{Ca}=-{i}_{Lb}$$

12 $${i}_{Cb}=-{i}_{0}$$

13 $${v}_{0}={v}_{cb}=-{i}_{0}R=-{i}_{Cb}R$$

The dynamic equations of State-2 are given below:

14 $$\left\{\begin{array}{c}\frac{{di}_{La}}{dt}=\frac{{v}_{a}}{La} \left({\delta }_{2}t\right)\\ \frac{{di}_{Lb}}{dt}= \frac{{v}_{{C}_{a}}}{Lb}{(\delta }_{2}t)\\ \begin{array}{c}\frac{{dv}_{{C}_{a}}}{dt}=\frac{{i}_{{L}_{b}}}{{C}_{a}}({\delta }_{2}t)\\ \frac{{dv}_{{C}_{b}}}{dt}=\frac{{v}_{o}}{{RC}_{b}}({\delta }_{2}t)\end{array}\end{array}\right.$$

15 $$\left[\begin{array}{c}\frac{{di}_{La}}{dt}\\ \frac{{di}_{Lb}}{dt}\\ \frac{{dv}_{Ca}}{dt}\\ \frac{{dv}_{Cb}}{dt}\end{array}\right]=\left[\begin{array}{cccc}0& 0& 0& 0\\ 0& 0& \frac{1}{{L}_{b}}& 0\\ 0& -\frac{1}{{C}_{a}}& 0& 0\\ 0& 0& 0& -\frac{1}{{RC}_{b}}\end{array}\right]\left[\begin{array}{c}{i}_{La}\\ {i}_{Lb}\\ {v}_{Ca}\\ {v}_{Cb}\end{array}\right]+\left[\begin{array}{cc}0& \frac{1}{{L}_{a}}\\ 0& 0\\ 0& 0\\ 0& 0\end{array}\right]\left[\begin{array}{c}{v}_{a}\\ {v}_{b}\end{array}\right]$$

16 $${v}_{o }=\left[\begin{array}{cccc}0& 0& 0& 1\end{array}\right] \left[\begin{array}{c}{i}_{La}\\ {i}_{Lb}\\ {v}_{Ca}\\ {v}_{Cb}\end{array}\right]+0$$

The suggested system exhibits a fourth-order configuration, and the state-space model can be formulated by employing the dynamic equations corresponding to State-2 are shown in Eqs. (15) and (16). with the inductor currents ${i}_{La}$ and ${i}_{Lb}$ and capacitor voltages ${v}_{Ca}$ and ${v}_{Cb}$ as the state variables.

#### State 3

In State–3, switches ${M}_{3}$ and ${M}_{4}$ are in the ON condition while ${M}_{1}$, ${M}_{2}$, Diode a, and Diode b are in the Inactive condition. During the operating phase from ${t}_{2}$ to ${t}_{3}$, equivalent to $\left({\delta }_{3 }\right)t$, as shown in Fig. 6. the converter exhibits specific characteristics. The equivalent circuit for this particular state of operation is presented in Fig. 4. The inductors ${L}_{a}$ and ${L}_{b}$ initiate the charging process, with the inductor currents ${i}_{La}$ and ${i}_{Lb}$ have slopes that are $\frac{{v}_{a}+{v}_{b}}{La}$ and $\frac{{v}_{Ca}}{Lb}$, respectively. The inductor current ${i}_{La}$ and ${i}_{Lb} reach \mathrm{their maximum values denoted as }{i}_{La}$ max and ${i}_{Lb}$ max. Simultaneously, the energy stored in capacitor ${C}_{b}$ begins to discharge through the load resistance R, capacitor voltage ${v}_{Cb}$ have slope that given by $\frac{-{v}_{o}}{RCb}$. Additionally, capacitors ${C}_{a}$ undergoes discharge, assisting ${L}_{b}$, with the slopes of the capacitor voltages ${v}_{Ca}$ determined by $\frac{-{i}_{Lb}}{Ca}$.

Applying KVL and KCL to the circuit shown in Fig. 4, the equations presented in (17) to (21) can be derived.

17 $${v}_{La}={v}_{a}+{v}_{b}$$

18 $${v}_{Lb}={v}_{ca}$$

19 $${i}_{Ca}=-{i}_{Lb}$$

20 $${i}_{Cb}=-{i}_{0}$$

21 $${v}_{0}={v}_{cb}=-{i}_{0}R=-{i}_{Cb}R$$

The dynamic equations of State–3 are given below:

22 $$\left\{\begin{array}{c}\frac{{di}_{La}}{dt}=\frac{{v}_{a}+{v}_{b}}{La} \left({\delta }_{3}t\right)\\ \frac{{di}_{Lb}}{dt}= \frac{{v}_{{C}_{a}}}{Lb}{(\delta }_{3}t)\\ \begin{array}{c}\frac{{dv}_{{C}_{a}}}{dt}=\frac{{i}_{{L}_{b}}}{{C}_{a}}({\delta }_{3}t)\\ \frac{{dv}_{{C}_{b}}}{dt}=\frac{{v}_{o}}{{RC}_{b}}({\delta }_{3}t)\end{array}\end{array}\right.$$

The suggested system exhibits a fourth-order configuration, and the state-space model can be formulated by employing the dynamic equations corresponding to State-3 are shown in Eqs. (23) and (24). with the inductor currents ${i}_{La}$ and ${i}_{Lb}$ and capacitor voltages ${v}_{Ca}$ and ${v}_{Cb}$ as the state variables.

23 $$\left[\begin{array}{c}\frac{{di}_{La}}{dt}\\ \frac{{di}_{Lb}}{dt}\\ \frac{{dv}_{Ca}}{dt}\\ \frac{{dv}_{Cb}}{dt}\end{array}\right]=\left[\begin{array}{cccc}0& 0& 0& 0\\ 0& 0& \frac{1}{{L}_{b}}& 0\\ 0& -\frac{1}{{C}_{a}}& 0& 0\\ 0& 0& 0& -\frac{1}{{RC}_{b}}\end{array}\right]\left[\begin{array}{c}{i}_{La}\\ {i}_{Lb}\\ {v}_{Ca}\\ {v}_{Cb}\end{array}\right]+\left[\begin{array}{cc}\frac{1}{{L}_{a}}& \frac{1}{{L}_{a}}\\ 0& 0\\ 0& 0\\ 0& 0\end{array}\right]\left[\begin{array}{c}{v}_{a}\\ {v}_{b}\end{array}\right]$$

24 $${v}_{o }=\left[\begin{array}{cccc}0& 0& 0& 1\end{array}\right] \left[\begin{array}{c}{i}_{La}\\ {i}_{Lb}\\ {v}_{Ca}\\ {v}_{Cb}\end{array}\right]+0$$

#### State-4

In State–4, Diode a and Diode b are in the ON condition, while ${M}_{1}$, ${M}_{2}$, ${M}_{3}$, and ${M}_{4}$ are in the Inactive condition. During the operating phase from ${t}_{3}$ to ${t}_{4}$, equivalent to $\left({1-\delta }_{1 }-{\delta }_{2 }-{\delta }_{3 }\right)t$, as illustrated in Fig. 6. the converter exhibits specific characteristics. The equivalent circuit for this particular state of operation is presented in Fig. 5. In this interval, the inductors ${L}_{a}$ and ${L}_{b}$ initiate the discharging process, with the slopes of the inductor currents ${i}_{La}$ and ${i}_{Lb}$ determined by $\frac{-{v}_{ca}-{v}_{cb}}{La}$ and $-\frac{{v}_{Cb}}{Lb}$, respectively. ${i}_{La}$ reaches its minimum value ${i}_{La}$ min from its maximum value ${i}_{La}$ max. while ${i}_{Lb}$ reaches its minimum value ${i}_{Lb}$ min from its maximum value ${i}_{Lb}$ max. Simultaneously, capacitors ${C}_{a}$ and ${C}_{b}$ begin to charge with the assistance of ${L}_{a}$ and ${L}_{b}$, with the slopes of the capacitor voltages ${v}_{Ca}$ and ${v}_{Cb}$ given by $\frac{{i}_{La}}{Ca}$ and $\frac{{i}_{La}}{Cb}+\frac{{i}_{Lb}}{Cb}-\frac{{v}_{0}}{R{C}_{b}}$,respectively.

Applying KVL and KCL to the circuit shown in Fig. 5, the equations presented in (25) to (28) can be derived.

25 $${v}_{La}={-v}_{Ca}+{-v}_{cb}$$

26 $${v}_{Lb}={-v}_{cb}$$

27 $${i}_{La}={i}_{Ca}$$

28 $${i}_{Lb}={i}_{0}+{i}_{Cb}-{i}_{La}$$

The dynamic equations of State-4 are given below:

29 $$\left\{\begin{array}{c}\frac{{di}_{La}}{dt}=\frac{{-v}_{{c}_{a}}-{v}_{{c}_{b}}}{La} \left({1-\delta }_{1 }-{\delta }_{2 }-{\delta }_{3 }\right)t\\ \frac{{di}_{Lb}}{dt}= -\frac{{v}_{{C}_{b}}}{Lb}({1-\delta }_{1 }-{\delta }_{2 }-{\delta }_{3 })t\\ \begin{array}{c}\frac{{dv}_{{C}_{a}}}{dt}=\frac{{i}_{{L}_{b}}}{{C}_{a}}({1-\delta }_{1 }-{\delta }_{2 }-{\delta }_{3 })t\\ \frac{{dv}_{{C}_{b}}}{dt}=\frac{{i}_{{L}_{a}}}{{C}_{b}}+\frac{{i}_{{L}_{b}}}{{C}_{b}}-\frac{{v}_{o}}{{RC}_{b}}({1-\delta }_{1 }-{\delta }_{2 }-{\delta }_{3 })t\end{array}\end{array}\right.$$

The suggested system exhibits a fourth-order configuration, and the state-space model can be formulated by employing the dynamic equations corresponding to State-4 are shown in Eqs. (30) and (31). with the inductor currents ${i}_{La}$ and ${i}_{Lb}$ and capacitor voltages ${v}_{Ca}$ and ${v}_{Cb}$ as the state variables.

30 $$\left[\begin{array}{c}\frac{{di}_{La}}{dt}\\ \frac{{di}_{Lb}}{dt}\\ \frac{{dv}_{Ca}}{dt}\\ \frac{{dv}_{Cb}}{dt}\end{array}\right]=\left[\begin{array}{cccc}0& 0& -\frac{1}{{L}_{a}}& -\frac{1}{{L}_{a}}\\ 0& 0& 0& -\frac{1}{{L}_{b}}\\ \frac{1}{{C}_{a}}& 0& 0& 0\\ \frac{1}{{C}_{b}}& \frac{1}{{C}_{b}}& 0& -\frac{1}{{RC}_{b}}\end{array}\right]\left[\begin{array}{c}{i}_{La}\\ {i}_{Lb}\\ {v}_{Ca}\\ {v}_{Cb}\end{array}\right]+\left[\begin{array}{cc}0& 0\\ 0& 0\\ 0& 0\\ 0& 0\end{array}\right]\left[\begin{array}{c}{v}_{a}\\ {v}_{b}\end{array}\right]$$

31 $${v}_{o }=\left[\begin{array}{cccc}0& 0& 0& 1\end{array}\right] \left[\begin{array}{c}{i}_{La}\\ {i}_{Lb}\\ {v}_{Ca}\\ {v}_{Cb}\end{array}\right]+0$$

## Analysis and design

### Average large signal model

There is a non-linear circuit design in the recommended configuration. The typical large-signal modeling method considers non-linearities and the impact of the real voltages in the circuit. As a result, the results obtained closely agree with the physical circuit's behaviour. The State-space illustration is a mathematical model that describes the dynamic behaviour of the system is as follows

$$\dot{x}=Ax+Bu$$

$$y=Cx+Du$$

A, B, C, and D are matrices representing the system dynamics, input–output relation, output observation, and feedforward components, respectively.

From Eqs. (7), (8), (15), (16), (23), (24), (29) and (30)

32 $$\left[\begin{array}{c}\frac{\widehat{{di}_{La}}}{dt}\\ \frac{\widehat{{di}_{Lb}}}{dt}\\ \frac{\widehat{{dv}_{Ca}}}{dt}\\ \frac{\widehat{{dv}_{Cb}}}{dt}\end{array}\right]=\left[\left[\begin{array}{cccc}0& 0& 0& 0\\ 0& 0& \frac{1}{Lb}& 0\\ 0& -\frac{1}{Ca}& 0& 0\\ 0& 0& 0& -\frac{1}{RCb}\end{array}\right]\left(\delta 1 + \delta 2 + \delta 3\right)+\left[\begin{array}{cccc}0& 0& -\frac{1}{La}& -\frac{1}{La}\\ 0& 0& 0& -\frac{1}{Lb}\\ \frac{1}{Ca}& 0& 0& 0\\ \frac{1}{Cb}& \frac{1}{Cb}& 0& -\frac{1}{RCb}\end{array}\right]\left(1-\delta 1- \delta 2- \delta 3\right)\right]\left[\begin{array}{c}{i}_{La}\\ {i}_{Lb}\\ {v}_{Ca}\\ {v}_{Cb}\end{array}\right]+\left[\begin{array}{cc}\frac{1}{La}\left(\delta 1+\delta 2\right)& \frac{1}{La}\left(\delta 1+\delta 2\right)\\ 0& 0\\ 0& 0\\ 0& 0\end{array}\right] \left[\begin{array}{c}{v}_{a}\\ {v}_{b}\end{array}\right]$$

33 $${v}_{o }=\left[\begin{array}{cccc}0& 0& 0& 1\end{array}\right] \left[\begin{array}{c}{i}_{La}\\ {i}_{Lb}\\ {v}_{Ca}\\ {v}_{Cb}\end{array}\right]+0$$

By comparing above Equations with general state-space representation as depicted in matrices A, B, and C the computations for matrices A, B, and C follow Eqs. (34) to (36), whereas matrix D remains a zero matrix.

34 $$\mathrm{A }=\left[\begin{array}{cccc}0& 0& 0& 0\\ 0& 0& \frac{1}{Lb}& 0\\ 0& -\frac{1}{Ca}& 0& 0\\ 0& 0& 0& -\frac{1}{RCb}\end{array}\right](\updelta 1 +\updelta 2 +\updelta 3) + \left[\begin{array}{cccc}0& 0& -\frac{1}{La}& -\frac{1}{La}\\ 0& 0& 0& -\frac{1}{Lb}\\ \frac{1}{Ca}& 0& 0& 0\\ \frac{1}{Cb}& \frac{1}{Cb}& 0& -\frac{1}{RCb}\end{array}\right](1 -\updelta 1 -\updelta 2 -\updelta 3)$$

35 $$\mathrm{B }=\left[\begin{array}{cc}\frac{1}{La}\left(\delta 1+\delta 2\right)& \frac{1}{La}\left(\delta 1+\delta 2\right)\\ 0& 0\\ 0& 0\\ 0& 0\end{array}\right]$$

36 $$\mathrm{C }=[\begin{array}{cccc}0& 0& 0& 1\end{array}]$$

### Small signal model

Small-signal models are very useful for analysing the behaviour of electronic circuits. They allow us to use linear circuit analysis techniques to analyse circuits containing nonlinear devices. This makes it much easier to design and analyse electronic circuits.

The small-signal model is then created by replacing the nonlinear device with its linearized equivalent circuit. This equivalent circuit typically consists of linear elements, such as resistors, capacitors, and voltage sources.

To analyse how this circuit responds to small changes, we can create a simplified model. This simplified model introduces small adjustments to the original circuit's control settings, internal conditions, and external inputs. Normally, these variables consist of a constant value (DC component) and a tiny variation around that value. By focusing on these small variations, we can analyse how the circuit behaves near its normal operating point

37 $$\left\{\begin{array}{c}{v}_{a}={V}_{a}+{\widehat{v}}_{a }\\ {v}_{b}={V}_{b}+{\widehat{v}}_{b}\\ { v}_{ca} ={V}_{ca}+{\widehat{v}}_{ca}\\ { v}_{cb}={V}_{cb}+{\widehat{v}}_{cb}\\ { i}_{La}={I}_{La}+{\widehat{i}}_{La}\\ {i}_{Lb}={I}_{Lb}+{\widehat{i}}_{Lb}\\ {\delta }_{1}={d}_{1}+{\widehat{\delta }}_{1}\\ {\delta }_{2}={d}_{2}+{\widehat{\delta }}_{2}\\ {\delta }_{3}={d}_{3}+{\widehat{\delta }}_{3}\end{array}\right.$$

where ${V}_{a}$ and ${V}_{b}$ stand for the DC source 1 and DC source 2's respective steady-state (DC element) voltages. Respectively. Additionally, the small-signal duty cycles of States 1 through 3 are represented by ${\widehat{\delta }}_{1}$ to ${\widehat{\delta }}_{3}$.${V}_{Ca}$ and ${V}_{Cb}$ denote the steady-state voltages across ${C}_{a}$ and ${C}_{b}$, respectively. Similarly, ${I}_{La}$ and ${I}_{Lb}$ represent the steady-state current flowing through ${L}_{a}$ and ${L}_{b}$. Additionally, ${d}_{1}$ to ${d}_{3}$ correspond to the steady-state duty cycle of State–1 to State–3. Furthermore, ${\widehat{i}}_{La}$ and ${\widehat{i}}_{Lb}$ are the small-signal current flowing through ${L}_{a}$ and ${L}_{b}$, while the small-signal (perturbation component) voltages of the DC source 1 and DC source 2 are represented by ${\widehat{v}}_{a}$ and ${\widehat{v}}_{b}$ Lastly, ${\widehat{v}}_{ca}$ and ${\widehat{v}}_{cb}$ denote the small-signal voltages across ${C}_{a}$ and ${C}_{b}$ ,respectively.

General state-space representation

38 $$\left\{\begin{array}{c}\dot{\widehat{x}}=\widehat{A}\widehat{x}+\widehat{B}\widehat{u}\\ \widehat{y}=\widehat{C}\widehat{x}+\widehat{D}\widehat{u}\end{array}\right.$$

Introducing perturbations into Eqs. (34)–(36), the resultant state-space illustration is expressed by the following Eqs. (39) and (40)

39 $$\left[\begin{array}{c}\frac{\widehat{{di}_{La}}}{dt}\\ \frac{\widehat{{di}_{Lb}}}{dt}\\ \frac{\widehat{{dv}_{Ca}}}{dt}\\ \frac{\widehat{{dv}_{Cb}}}{dt}\end{array}\right]=\left[\left[\begin{array}{cccc}0& 0& 0& 0\\ 0& 0& \frac{1}{Lb}& 0\\ 0& -\frac{1}{Ca}& 0& 0\\ 0& 0& 0& -\frac{1}{RCb}\end{array}\right] \left(d1 + d2 + d3\right) +\left[\begin{array}{cccc}0& 0& -\frac{1}{La}& -\frac{1}{La}\\ 0& 0& 0& -\frac{1}{Lb}\\ \frac{1}{Ca}& 0& 0& 0\\ \frac{1}{Cb}& \frac{1}{Cb}& 0& -\frac{1}{RCb}\end{array}\right] (1 - d1 - d2 - d3)\right]\left[\begin{array}{c}\widehat{{i}_{La}}\\ \widehat{{i}_{Lb}}\\ \widehat{{v}_{Ca}}\\ \widehat{{v}_{Cb}}\end{array}\right]+\dots +\left[\begin{array}{ccccc}\frac{Vca+Vcb+Va}{La}& \frac{Vca+Vcb+Vb}{La}& \frac{Vca+Vcb+Vb+Va}{La}& \frac{d1 + d3}{La}& \frac{d2 + d3}{La}\\ \frac{Vca+Vcb}{Lb}& \frac{Vca+Vcb}{Lb}& \frac{Vca+Vcb}{Lb}& 0& 0\\ \frac{{I}_{La}+{I}_{Lb}}{Ca}& \frac{{I}_{La}+{I}_{Lb}}{Ca}& \frac{{I}_{La}+{I}_{Lb}}{Ca}& 0& 0\\ \frac{{I}_{La}+{I}_{Lb}}{Ca}& \frac{{I}_{La}+{I}_{Lb}}{Ca}& \frac{{I}_{La}+{I}_{Lb}}{Ca}& 0& 0\end{array}\right] \left[\begin{array}{c}\widehat{{\delta }_{1 }}\\ \widehat{{\delta }_{2 }}\\ \widehat{{\delta }_{3 }}\\ \begin{array}{c}{\widehat{v}}_{a}\\ {\widehat{ \, v}}_{b}\end{array}\end{array}\right]$$

40 $${\widehat{v}}_{o }=\left[\begin{array}{cccc}0& 0& 0& 1\end{array}\right] \left[\begin{array}{c}\widehat{{i}_{La}}\\ \widehat{{i}_{Lb}}\\ \widehat{{v}_{Ca}}\\ \widehat{{v}_{Cb}}\end{array}\right]+0$$

By comparing Equation general state-space illustration as shown in Eq. (38) with the Eqs. (39) and (40). The state vector matrix ${\hat{\text{x}}}$ represents the small-signal model, the system matrix ${\hat{\text{A}}}$, the first derivative of the small-signal state vector matrix ${\hat{\text{x}}}$, the input matrix ${\hat{\text{B}}}$, the output matrix ${\hat{\text{C}}}$ and the feedforward matrix ${\hat{\text{D}}}$, The input vector matrix for the small-signal model is denoted as ${\hat{\text{u}}}$, and the output vector matrix is represented by ${\hat{\text{y}}}$ respectively, The characteristic equation is

41 $$\left|SI- \widehat{A}\right|= 0$$

the 4 × 4 identity matrix, designated as I, and the Laplace transform variable S.

42 $${(S)}^{4}+{(S)}^{3}\left[\frac{1}{\left(R {C}_{b}\right) }\right]+{(S)}^{2}\left[\frac{{\left(d1 + d2 + d3\right)}^{2}}{\left({C}_{a}{L}_{b}\right) }+\frac{{\left(1 - d1 - d2 - d3\right)}^{2}}{\left({C}_{b}{L}_{b}\right) }+\frac{{\left(1 - d1 - d2 - d3\right)}^{2}}{\left({C}_{a}{L}_{a}\right) }++\frac{{\left(1 - d1 - d2 - d3\right)}^{2}}{\left({C}_{a}{L}_{a}\right) }\right]+{\text{S}}\left[\frac{{\left(d1 + d2 + d3\right)}^{2}}{\left(R {C}_{a}{C}_{b}{L}_{b}\right) }+\frac{{\left(1 - d1 - d2 - d3\right)}^{2}}{\left(R {C}_{a}{C}_{b}{L}_{a}\right) }\right]+\frac{{(1 - d1 - d2 - d3)}^{2}}{({C}_{a}{C}_{b}{L}_{a}{L}_{b}) }=0$$

Compare Eq. (39) with the equation below:

43 $${(S)}^{4}+ {(S)}^{3}{\text{a}}+{(S)}^{2}b+Sc+d=0$$

The Routh-Hurwitz stability criterion (RHSC) offers a method to determine the stability of a linear system without directly calculating its poles. Instead, RHSC relies on the coefficients of the characteristic equation. Given that the system under consideration is of fourth order, employing the R–H stability criterion proves advantageous over a pole-zero plot. This strategy circumvents the challenge of dealing with zeros and poles in higher-order systems. Below is the R–H stability criterion Table. 2 illustrating the stability analysis for the proposed configuration.

**Table 2:** R–H stability criterion.

${S}^{4}$	1	b	d
${S}^{3}$	a	C	0
${S}^{2}$	$\frac{ab-c}{a}$	d	0
${S}^{1}$	$\frac{abc-{c}^{2}-{a}^{2}d}{ab-c}$	0	0
${S}^{0}$	d	0	0

## Stability validation

Stability verification is accomplished by examining the parameters in Table. 4 and solving for the coefficients in Characteristic Eq. (42). This calculation yields the coefficients of the characteristic equation, allowing us to assess the system's stability. The below is shown that characteristics equation of the proposed system.

44 $${(S)}^{4}+{(S)}^{3}619.175+{(S)}^{2}\mathrm{13,346,680}+\left(S\right)\mathrm{54,232,122,135}+\mathrm{16,700,050,066,750}=0$$

R–H stability criterion.

**Table:** 

${S}^{4}$	1	$\mathrm{13,346,680}$	$\mathrm{16,700,050,066,750}$
${S}^{3}$	619	$\mathrm{54,232,122,135}$	0
${S}^{2}$	$\mathrm{4,585,432}$	$\mathrm{16,700,050,066,750}$	0
${S}^{1}$	$\mathrm{3,168,826,726}$	0	0
${S}^{0}$	16,700,050,066,750		0

### Steady-state modelling

Steady-state modelling of DC source 1 (${v}_{a}$) and DC source 2 (${v}_{b}$) hybrid system can be used to design the system parameters to achieve the desired output voltage and current, even under varying operating conditions. Consider that ${V}_{a}$ and ${V}_{b}$ are the steady-state DC source 1 and DC source 2 voltages, respectively. ${V}_{La}$ and ${V}_{Lb}$ are the voltage across in steady state ${L}_{a}$ and ${L}_{b}$, respectively, and ${IL}_{a}$ and ${IL}_{b}$ are the current flowing in steady-state through ${L}_{a}$ and ${L}_{b}$, respectively. ${V}_{Ca}$ and ${V}_{Cb}$ are the voltage across in steady state ${C}_{a}$ and ${C}_{b}$, respectively, and ${IC}_{a}$ and ${IC}_{b}$ are the current flowing in steady-state through ${C}_{a}$ and ${C}_{b}$, respectively. ${ID}_{a}$ and ${ID}_{b}$ are the current flowing in steady-state through diodes Diode a and Diode b. ${I}_{a}$ and ${I}_{b}$ are the current flowing in steady-state from the source DC source 1 and DC source 2, respectively.

From Eqs. (1)–(5), (10)–(13), (17)–(21) and (24)–(27),

Volt-Sec balance equation at La,

45 $${V}_{a}{d}_{1}T+{V}_{b}{d}_{2}T+\left({V}_{a}+{V}_{b}\right){d}_{3}T+\left(-{V}_{Ca}- {V}_{0}\right)\left(1-{d}_{1}-{d}_{2}-{d}_{3}\right)T=0$$

By simplifying the above equation

46 $${V}_{a}\left({d}_{1}+{d}_{3}\right)+{V}_{b}\left({d}_{2}+{d}_{3}\right)-{V}_{Ca}\left(1-{d}_{1}-{d}_{2}-{d}_{3}\right)-{V}_{0}\left(1-{d}_{1}-{d}_{2}-{d}_{3}\right)=0$$

Volt-Sec balance equation at Lb,

47 $${V}_{Ca}{d}_{1}T+{V}_{Ca}{d}_{2}T+{V}_{Ca}{d}_{3}T-{V}_{0}\left(1-{d}_{1}-{d}_{2}-{d}_{3}\right)T=0$$

By simplifying the above equation

48 $${V}_{Ca}= {V}_{0 } ( \frac{1-{d}_{1}-{d}_{2}-{d}_{3}}{{d}_{1}+{d}_{2}+{d}_{3}} )$$

Substitute Eq. (48) in Eq. (45) to determine the output voltage expression given by Eq. (49).

49 $${V}_{0}=\frac{{d}_{1}+{d}_{2}+{d}_{3}}{1-{d}_{1}-{d}_{2}-{d}_{3}} ({V}_{a}\left( {d}_{1}+{d}_{3}\right)+{V}_{a}\left({d}_{2}+{d}_{3}\right))$$

Amp-Sec balance equation at Ca,

50 $$-{I}_{Lb}{d}_{1}T-{I}_{Lb}{d}_{2}T-{I}_{Lb}{d}_{3}T+ {I}_{La}\left( 1-{d}_{1}-{d}_{2}-{d}_{3}\right)T=0$$

By simplifying the above equation

51 $$-{I}_{Lb}\left({d}_{1}+{d}_{2}+{d}_{3}\right)+{I}_{La}\left( 1-{d}_{1}-{d}_{2}-{d}_{3}\right)=0$$

Amp-Sec balance equation at C2,

52 $$-{I}_{0}{d}_{1}T-{I}_{0}{d}_{2}T-{I}_{0}{d}_{3}T+\left({I}_{La}+{I}_{Lb}-{I}_{0}\right) \left(1-{d}_{1}-{d}_{2}-{d}_{3}\right)T=0$$

By simplifying the above equation,

53 $$\left(1-{d}_{1}-{d}_{2}-{d}_{3}\right)+ {I}_{La}\left(1-{d}_{1}-{d}_{2}-{d}_{3}\right)={I}_{0}$$

By solving Eqs. (51) and (53),

54 $${I}_{Lb}= {I}_{0}= \frac{{V}_{0}}{R}$$

55 $${I}_{La}=\frac{{V}_{0}}{R} \frac{{d}_{1}+{d}_{2}+{d}_{3}}{1-{d}_{1}-{d}_{2}-{d}_{3}}$$

### Designing capacitors and inductors

The precise choice of capacitors and inductors is pivotal in shaping the system's performance, facilitating its operation in the specified conduction mode. The careful selection of these components allows for fine-tuning the system's characteristics, ensuring optimal functionality and adherence to the desired operational mode. This deliberate approach to capacitor and inductor selection significantly influences the overall performance and efficiency of the configuration.

The value of *La* is provided by Eq. (56).

56 $${L}_{a}= \frac{({V}_{a}\left({d}_{1}+{d}_{3}\right)+ {V}_{b}\left({d}_{2}+{d}_{3}\right)T}{\Delta {I}_{La}}$$

The change in ${I}_{La}$, denoted as ∆${I}_{La}$, can be expressed as given in Eq. (57).

57 $$\Delta {I}_{La}= {I}_{La max}- {I}_{La min}$$

The value of ${L}_{b}$ is provided by Equation ($58$)

58 $${L}_{b}= \frac{{V}_{0}\left(1-{d}_{1}-{d}_{2}-{d}_{3}\right)T}{\Delta {I}_{Lb}}$$

The change in ${I}_{Lb}$, denoted as ∆${I}_{Lb}$, can be expressed as given in Eq. (59).

59 $$\Delta {I}_{Lb}= {I}_{Lb max}- {I}_{Lb min}$$

The value of *Ca* is provided by Equation ($60$)

60 $${C}_{a}= \frac{{V}_{0}\left({d}_{1}+{d}_{2}+{d}_{3}\right)T}{\mathrm{R \Delta }{V}_{Ca}}$$

The change in ${V}_{Ca}$, denoted as ∆${V}_{Ca}$, can be expressed as given in Eq. (61).

61 $$\Delta {V}_{Ca}= {V}_{Ca\mathit{max} }- {V}_{Ca min}$$

The value of ${C}_{b}$ is given by Equation ($62$)

62 $${C}_{b}=\frac{{V}_{0}\left({d}_{1}+{d}_{2}+{d}_{3}\right)T}{\mathrm{R \Delta }{V}_{Cb}}$$

The change in ${V}_{Cb}$, denoted as ∆${V}_{Cb}$, can be expressed as given in Eq. (61).

63 $$\Delta {V}_{Cb}= {V}_{Cb\mathit{max} }- {V}_{Cb min}$$

### Efficiency and voltage stress calculations

#### Voltage stress calculations

The selection of electrical switches and diodes requires meticulous attention to voltage stress, a critical factor in ensuring optimal performance. The determination of voltage stresses is essential, particularly when a power electronic switch is in the off state. In this scenario, voltage stress is equivalent to the maximum magnitude of voltage across the switch.

The voltage stress of S_1_(V_VS1_) is given below.

64 $${V}_{VM1}=\mathit{max}\left(0, {V}_{a}-{V}_{b}, - {V}_{b}, {V}_{a}\right)={V}_{a}$$

The voltage stress of S_2_(V_VS2_) is given below.

65 $${V}_{VM2}=\mathit{max}\left( {V}_{b}-{V}_{a}, 0, - {V}_{a}, {V}_{b}\right)={V}_{b}$$

The voltage stress of S_3_(V_VS3_) is given below.

66 $${V}_{VM3} ={\text{max}}({V}_{b}, {V}_{a}, 0, {V}_{a}+{V}_{b})= {V}_{a}+{V}_{b}$$

The voltage stress of S_4_(V_VS4_) is given below

67 $${V}_{VM4}={\text{max}}\left(0, 0, 0, -{V}_{La}\right)= {V}_{La}={V}_{Ca}+{V}_{0}= \frac{1}{1-{d}_{1}-{d}_{2}-{d}_{3}}( {V}_{a}\left({d}_{1}+{d}_{3}\right)+{V}_{b}\left({d}_{2}+{d}_{3}\right) )$$

The voltage stress of Diode1(V_VSD1_) is given below.

68 $${V}_{VMDa}={\text{max}}(-{V}_{a},-{V}_{b}, -\left({V}_{a}+{V}_{b}\right), 0 )= {V}_{a}+{V}_{b}$$

The voltage stress of Diode2(V_VSD2_) is given below.

69 $${V}_{VMDb}={\text{max}}(-{V}_{Lb}-{V}_{0}, {-V}_{Lb}-{V}_{0}, - {V}_{Lb}-{V}_{0}, 0)= {V}_{Lb}+{V}_{0} = {V}_{C1}+{V}_{0}$$

#### Efficiency calculations

Efficiency is the ability to achieve a desired output with the least amount of input. Efficiency is important for several reasons. It can help to reduce costs, save time, and improve productivity. Therefore, the efficiency of the converter is

70 $$\%Efficiency= \frac{{P}_{out}}{ {P}_{in}}*100$$

#### Possible operating scenarios

The suggested configuration offers versatility by supporting operation in three distinct modes, each characterized by its unique output voltage equations, as outlined in Table 3. where ${D}_{4}$ represents the duty ratio associated with switch ${M}_{4}$, and, respectively, ${D}_{1}$, ${D}_{2}$, and ${D}_{3}$ are the duty ratios of switches ${M}_{1}$, ${M}_{2}$ and ${M}_{3}$, respectively.

**Table 3:** Operating cases of the proposed configuration.

Cases	DC source 1 (${{\text{v}}}_{{\text{a}}}$)	DC source 2 (${{\text{v}}}_{{\text{b}}}$)	Action	Output voltage (${{\text{v}}}_{0}$)
i	1	0	a single DC source 1 handling the load	$\frac{{{\text{V}}}_{1}\left({{\text{d}}}_{4}\right)}{1- {{\text{d}}}_{4}}$(${{\text{d}}}_{1}$ = 1)
ii	0	1	DC source 2 handling the load	$\frac{{{\text{V}}}_{2}\left({{\text{d}}}_{4}\right)}{1- {{\text{d}}}_{4}}$(${{\text{d}}}_{2}$ = 1)
iii	1	1	DC source 1 and DC source 2 handling the load the load	$\frac{({{\text{V}}}_{1}+{{\text{V}}}_{2})\left({{\text{d}}}_{4}\right)}{1- {{\text{d}}}_{4}}$(${{\text{d}}}_{3}$ = 1)

## Results

The resulting waveforms presented here serve as empirical evidence supporting our assertion of high gain and efficiency. This dynamic modelling approach enhances our understanding of the converter's operation, reinforcing our claim of superior performance across diverse scenarios. The projected converter is designed for an output power of 100 W, with an output voltage of 54 V. With input voltages of 12 V and 24 V, they can be produced at a duty ratio of 25%. The load resistance is calculated using the basic formula as 29.1 Ω. To reduce the converter size, it is advisable to take higher switching frequencies (f_s_), however, for the proposed simulation and design 50 kHz includes Two inductors and Two capacitors. With the considerable current and voltage ripples on the inductors and capacitors, respectively. The energy component values are calculated and are observed in Table 4.

**Table 4:** Simulation parameter values.

Component	Parameter	Specification
${V}_{a},{V}_{b}$	Input voltage	12 V, 24 V
${V}_{o}$	Output voltage	108 V
${P}_{o}$	Output power	100W
${f}_{s}$	Switching frequency	50,000 Hz
${R}_{o}$	Load resistor (R)	29.1 Ω
${d}_{1}$*, *${d}_{2}$*,*${d}_{3}$	Duty ratio	0.25
${d}_{4}$	Duty ratio	0.75
${L}_{a }, {L}_{b}$	Inductor	0.9 mH, 1.35 mH
${C}_{a }, {C}_{b}$	Capacitors	55.5 µF, 55.5 µF

### Theoretical calculations

We explore the theoretical calculations for the proposed converter, examining three distinct cases. For each case, the theoretical framework is summarized in the Table 5.

**Table 5:** Theoretical calculations for three different cases.

Cases	Input voltage (V)	Output voltage (V)
DC source 1	12	36
DC source 2	24	72
DC source 1 and DC source 2	36	108

### Simulation circuit for different cases

#### Case-1

**Figure 7:**
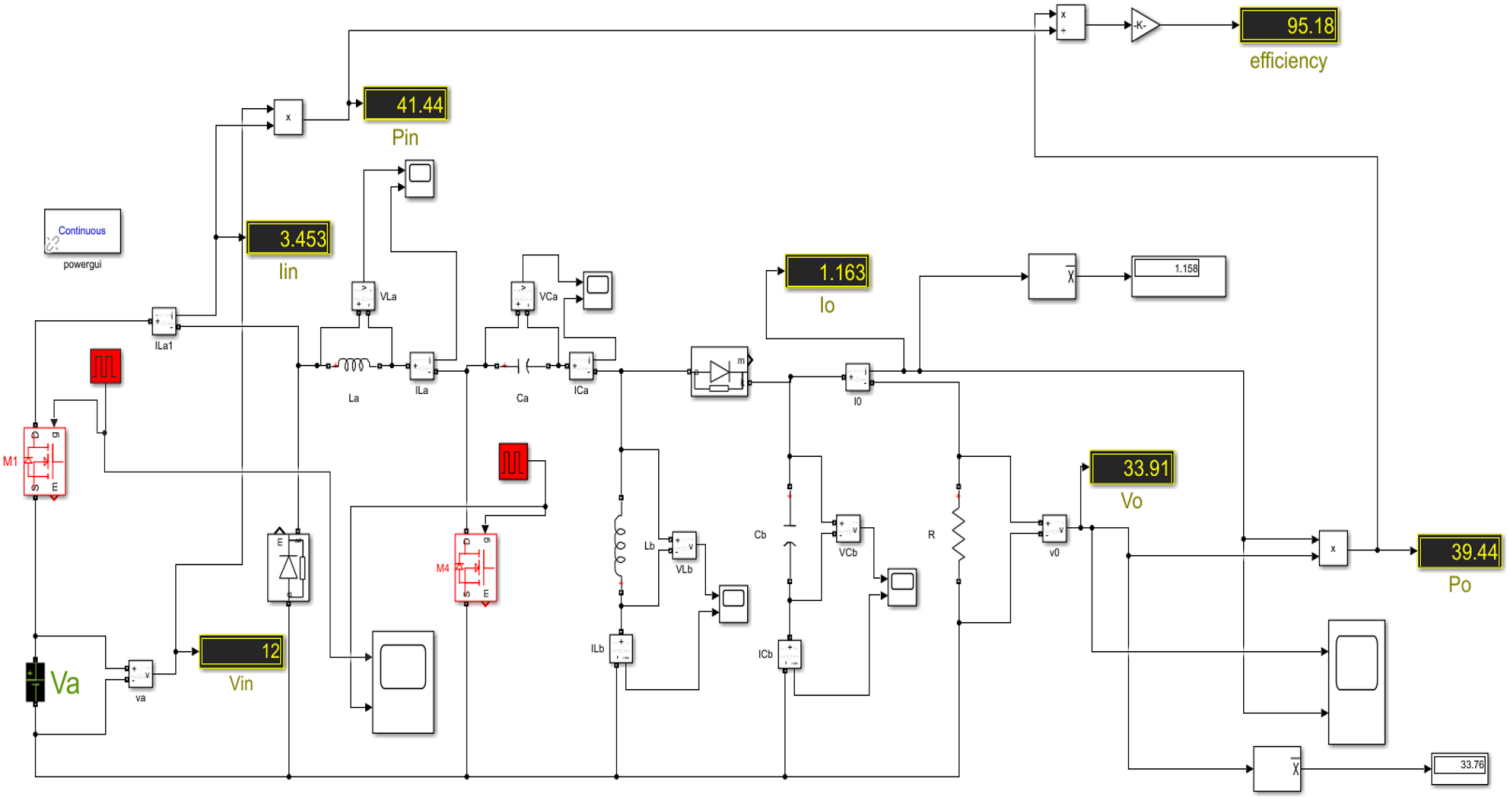
Simulink diagram designed for Case-1.

In this case, the DC source 1 acts as the primary source, providing an input voltage of 12 V and yielding an output voltage of 36 V. Switches ${M}_{1}$ and ${M}_{4}$ are both in the ON state, with a duty ratio of 0.25 for ${M}_{1}$ and 0.75 for ${M}_{4}$. The Simulink diagram for Case-1 is depicted in Fig. 7.

**Figure 8:**
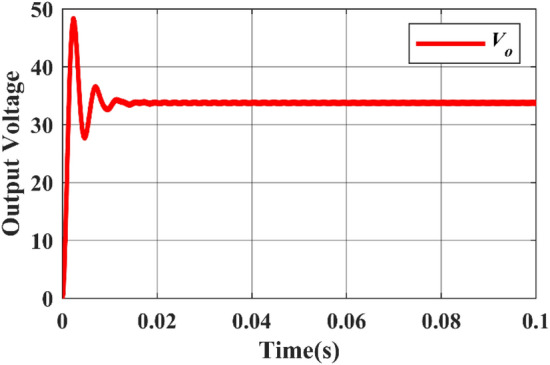
Simulated output voltage waveform.

**Figure 9:**
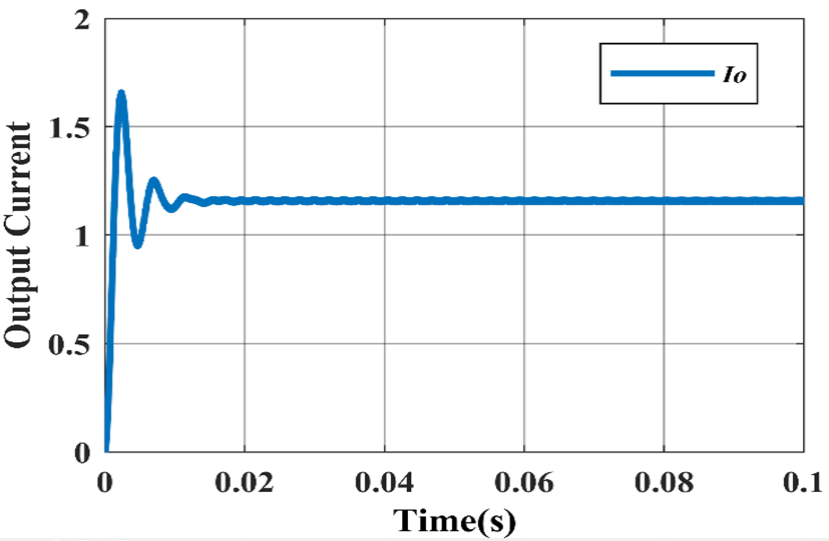
Simulated output current waveform.

While Figs. 8 and 9 depicts the corresponding output voltage and output current waveforms. From the waveforms, the rise time can be determined as 0.002 s and the settling time is 0.014 s.

#### Case-2

**Figure 10:**
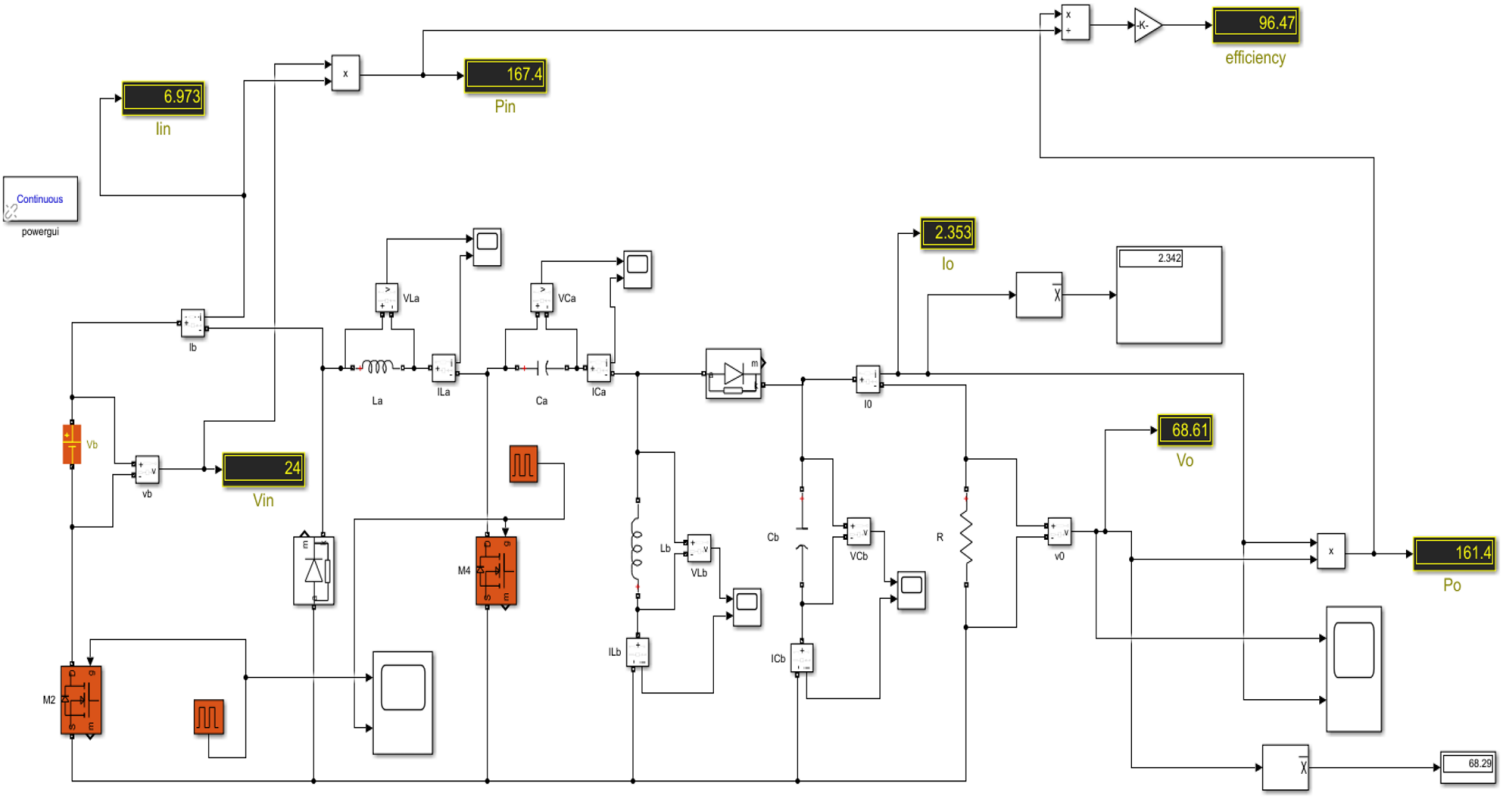
Simulink diagram for case-2.

In this case, the DC source 1 acts as the primary source, providing an input voltage of 24 V and yielding an output voltage of 72 V. Switches ${M}_{2}$ and ${M}_{4}$ are both in the ON state, with a duty ratio of 0.25 for *M*_*2*_ and 0.75 for ${M}_{4}$. The Fig. 10 displays the Simulink diagram for Case 2.

**Figure 11:**
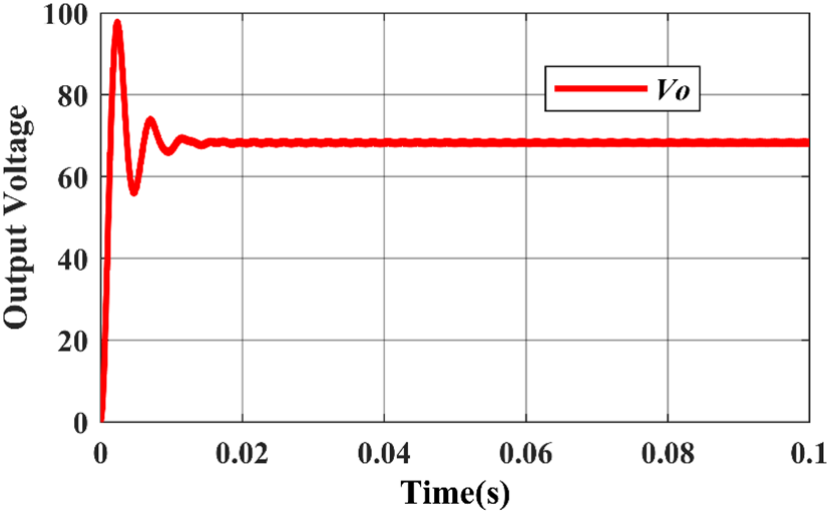
Simulated output voltage waveform.

**Figure 12:**
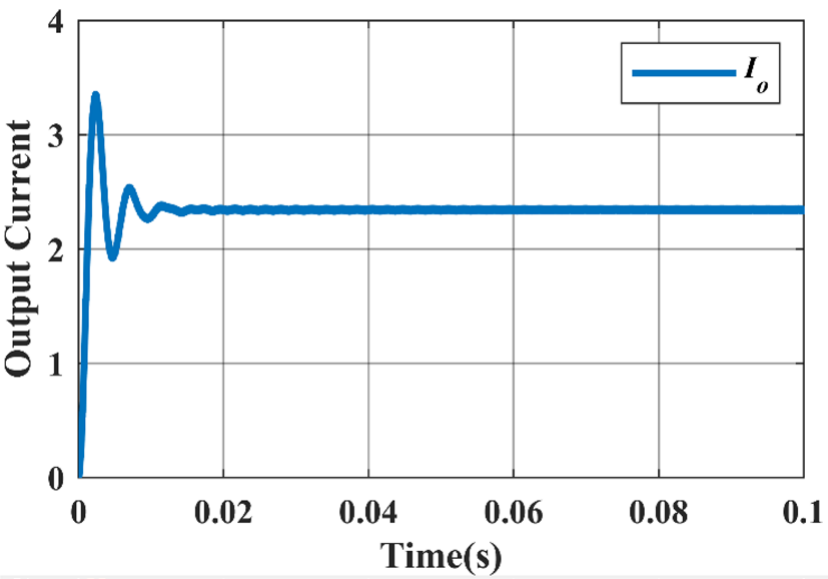
Simulated output current waveform.

While Figs. 11 and 12 depicts the corresponding output voltage and output current waveform.

#### Case-3

**Figure 13:**
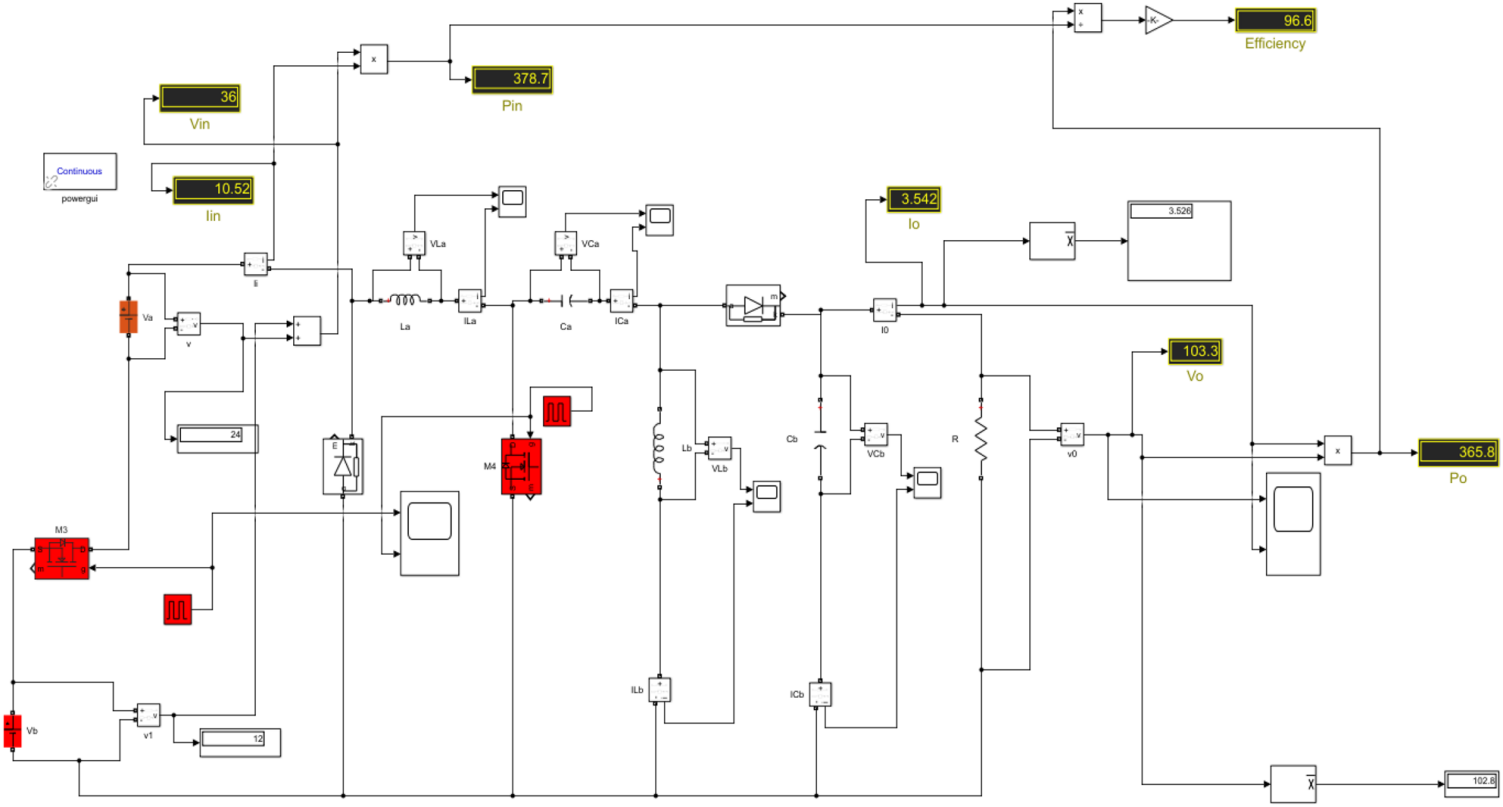
Simulink diagram for case-3.

**Figure 14:**
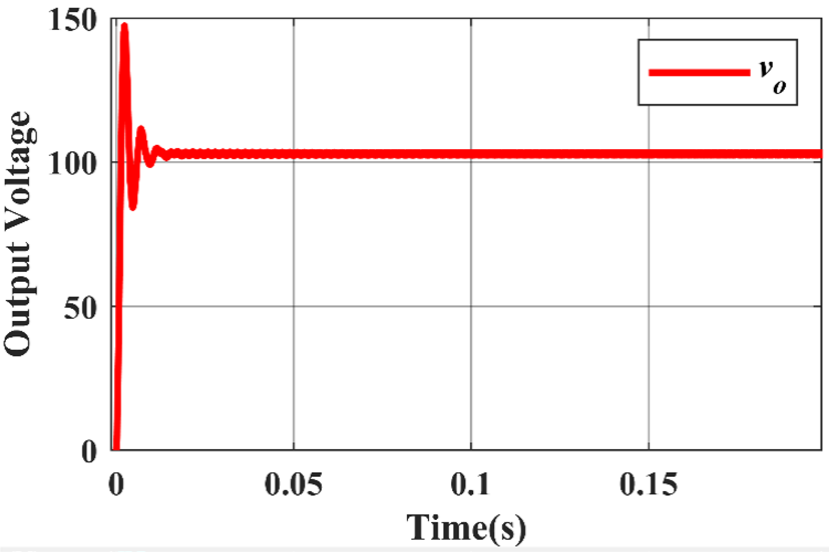
Simulated output voltage waveform.

**Figure 15:**
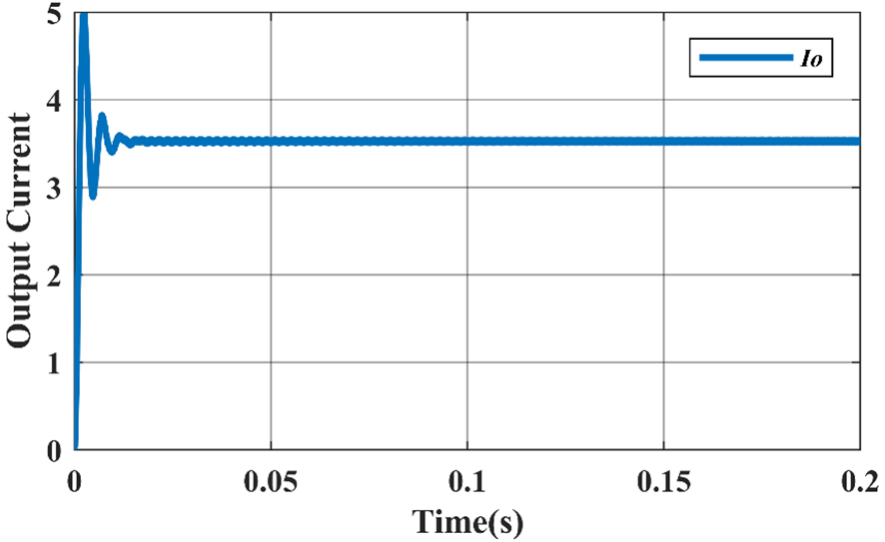
Simulated output current waveform.

In this case, the DC source 1 acts as the primary source, providing an input voltage of 36 V and yielding an output voltage of 108 V. Switches ${M}_{3}$ and ${M}_{4}$ are both in the ON state, with a duty ratio of 0.25 for ${M}_{3}$ and 0.75 for ${M}_{4}$. The Fig. 13 displays the Simulink diagram for Case 3. While Figs. 14 and 15 depict the corresponding output voltage and current waveform.

This Table 6 comprehensively compares the parameters of performance for each of the three instances, including input voltage, input current, input power, output voltage, output current, output power, efficiency, and ripple factor.

**Table 6:** Practical calculations for three different cases.

Cases	${V}_{in}$	${I}_{in}$	${P}_{in}$	${V}_{0}$	${I}_{0}$	${P}_{0}$	Efficiency (%)	Ripple factor
Case-1	12	3.447	41.36	33.6	1.163	39.46	95.39	0.91
Case-2	24	6.973	167.4	68.61	2.353	161.4	96.47	0.88
Case-3	36	10.52	378.7	103.3	3.542	365.8	96.6	0.86

**Figure 16:**
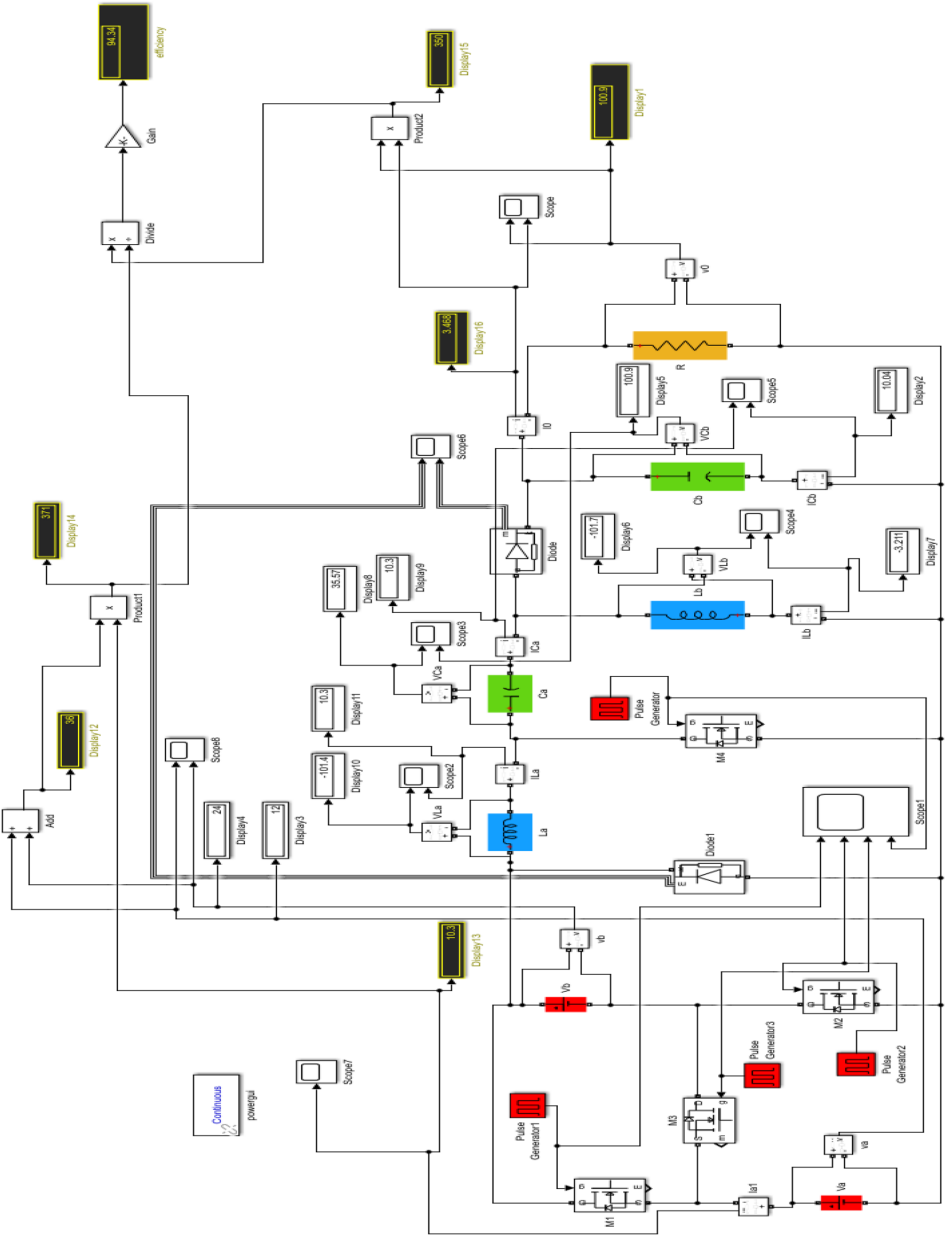
Simulink schematic for the proposed converter.

We have examined each of the three cases from the preceding discussion individually. The Simulink diagram presented visually represents the proposed converter, which incorporates inductors, capacitors, diodes, four switches with phase delay, and is powered by both DC source 1 and DC source 2. The Fig. 16 displays the Simulink diagram for proposed converter.

**Figure 17:**
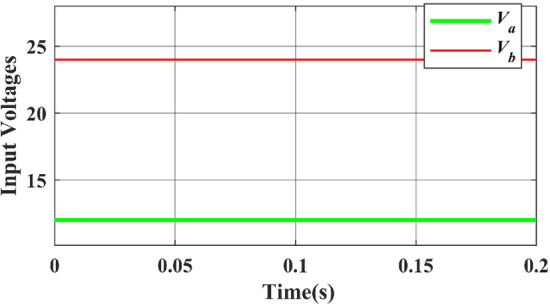
Simulated input DC voltages waveform.

**Figure 18:**
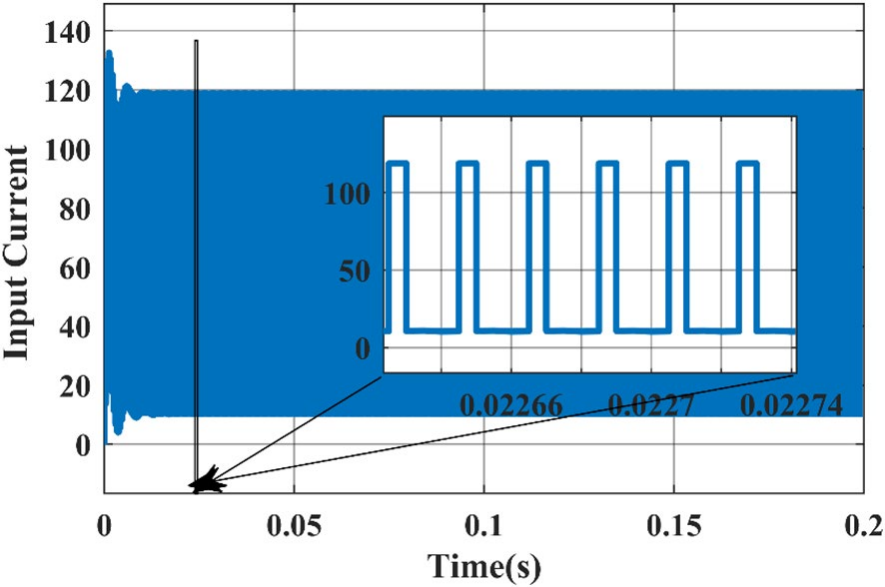
Simulated waveform of the input DC Current.

Figure 17 represents the input DC voltage waveform and Fig. 18 represents the input current plotted using MATLAB simulation. A 12 V & 24 V DC input voltage is considered when designing the proposed topology. Similarly, it can be observed that the input current waveform is continuous.

Figure 18 shows that the input current never reaches zero, indicating continuous current conduction from the input. The observed current interval from 1 to 200.

**Figure 19:**
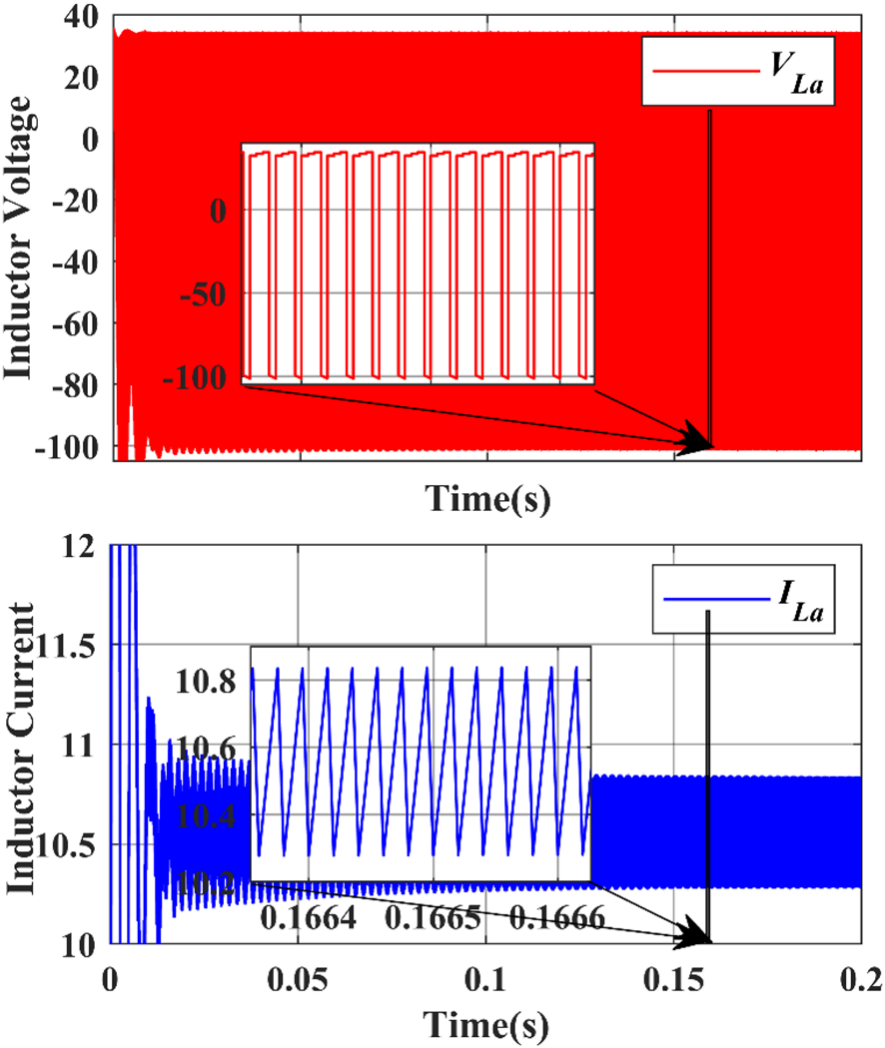
Simulation Inductor voltage (${{\text{V}}}_{{\text{La}}}$) & Inductor current (I_La_) waveforms.

**Figure 20:**
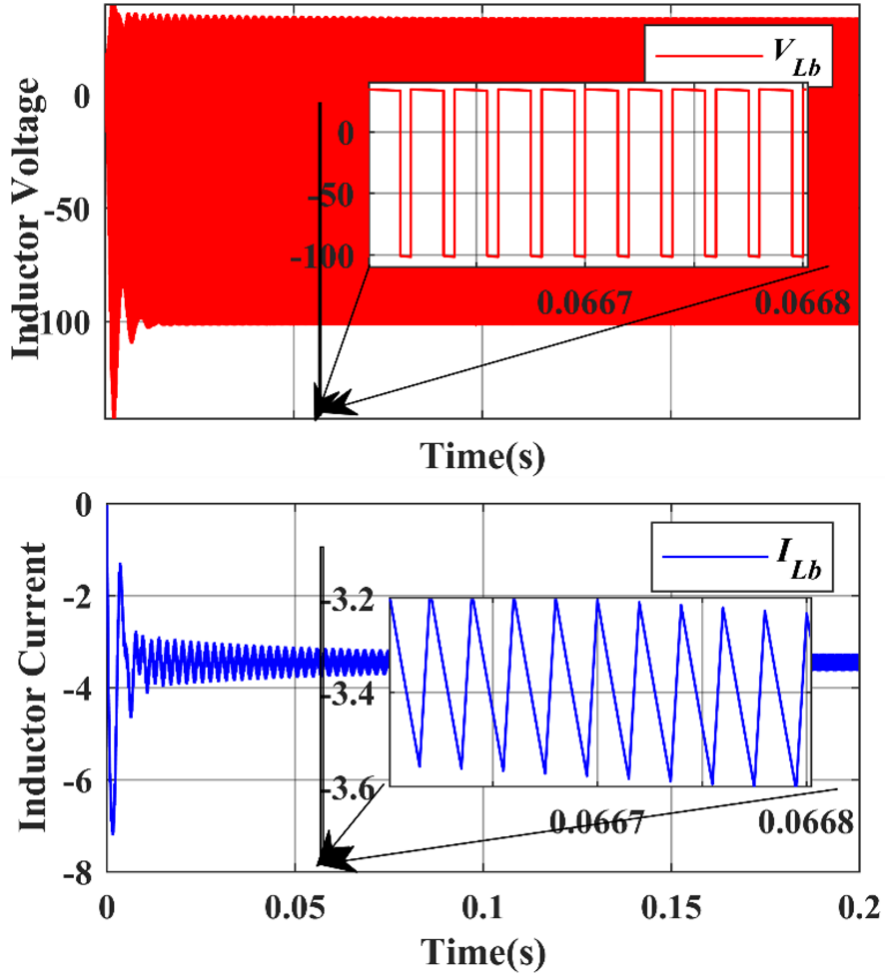
Simulation Inductor voltage (${{\text{V}}}_{{\text{Lb}}}$) & Inductor current (I_Lb_) waveforms.

The inductors ${L}_{a}$ & ${L}_{b}$ are charged when the active switches are in ON state and they will discharge their energy when the active switches are in OFF state. Figure 19 shows the simulated inductor (${L}_{a}$) current waveforms and inductor voltage waveforms, respectively under steady-state operation. Figure 20 shows the simulated inductor(b) current waveforms and inductor voltage waveforms under steady-state operation. The capacitor ${C}_{a}$ and ${C}_{b}$ discharge the energy when the active switches are turned on, and charges when the switch is turned off. The capacitor voltage waveforms can be observed in Figs. 23 and 24. The capacitor current waveforms can be observed in Figs. 25 and 26.

Under the steady state condition, the graph shows a stable output. Figure 19 shows the current from 10 to 12 during the time interval between 0.1664 and 0.1666, whereas the voltage from − 100 to 40 during the time interval between 0.1664 and 0.1666. Under the steady state condition, the graph shows a stable output. Figure 20 shows the current from − 8 to − 2 during the time interval between 0.0667 and 0.0668, whereas the voltage is from − 100 to 0 during the time interval between 0.0667 and 0.0668.

**Figure 21:**
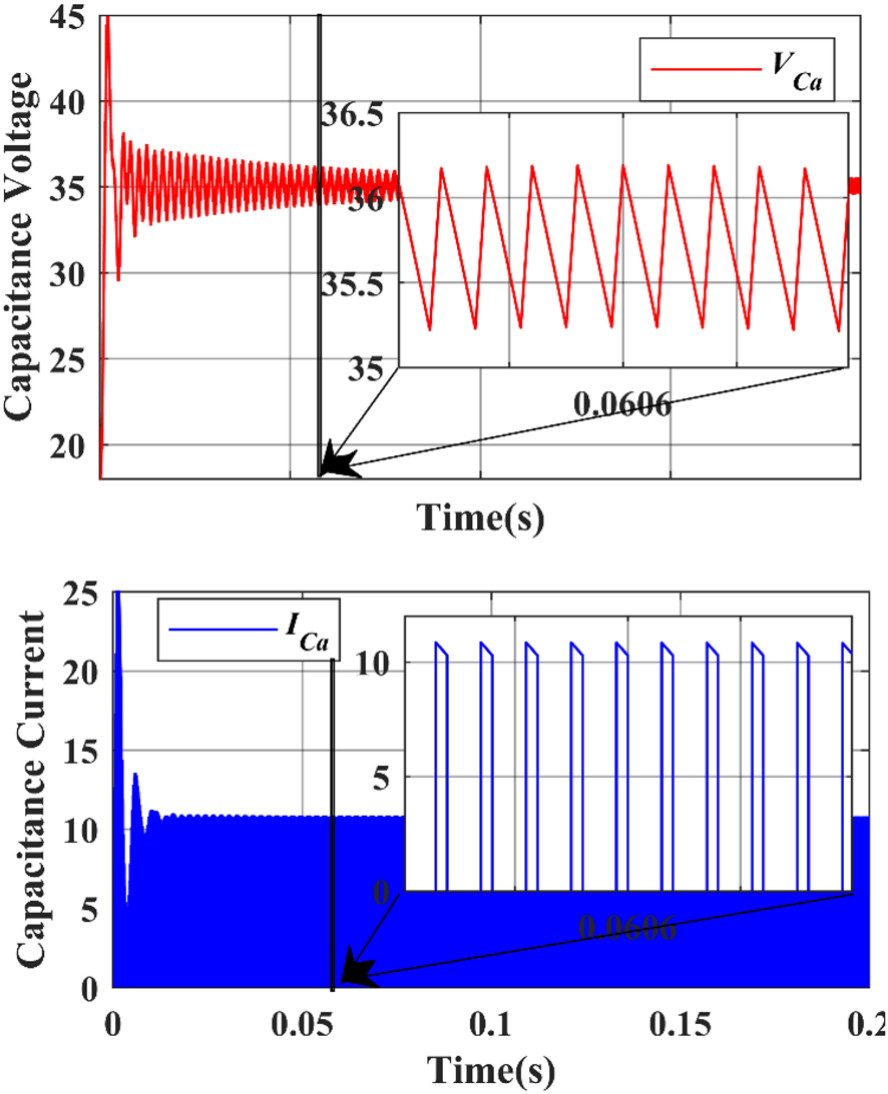
Simulation capacitor voltage (${{\text{V}}}_{{\text{Ca}}}$) & Capacitor current (${{\text{I}}}_{{\text{Ca}}}$) waveforms.

Under the steady state condition, the graph shows a stable output. Figure 21 shows the capacitor (${C}_{a}$) voltage from 20 to 45 during the time interval between 0.0606 and 0.0608, whereas the capacitor (${C}_{a}$) current from 0 to 25 during the time interval between 0.0606 and 0.0608.

**Figure 22:**
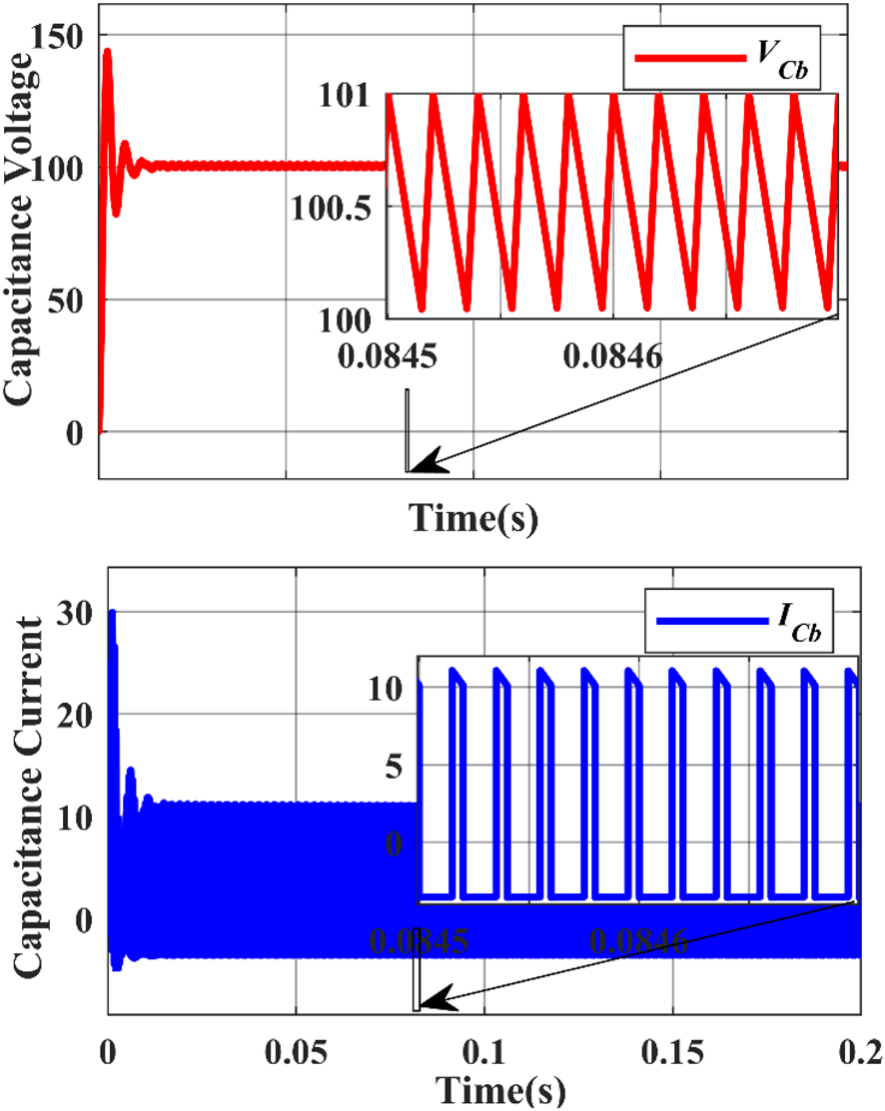
Simulation Capacitor voltage (${{\text{V}}}_{{\text{Cb}}}$) & Capacitor current (${{\text{I}}}_{{\text{Cb}}}$) waveforms.

Under the steady state condition, the graph shows a stable output. Figure 22 shows the capacitor (${C}_{b}$) voltage from 0 to 150 during the time interval between 0.0845 and 0.0846, whereas the capacitor (${C}_{b}$) current from 0 to 30 during the time interval between 0.0845 and 0.0846.

**Figure 23:**
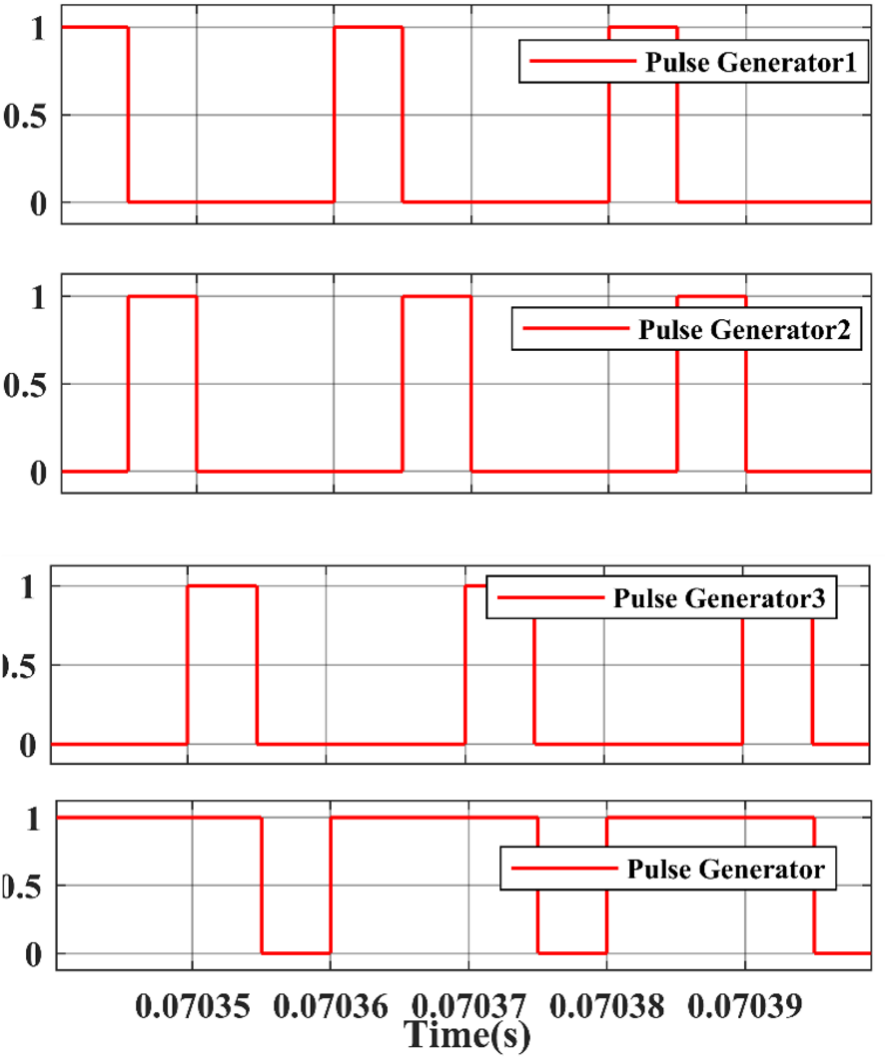
Simulation waveform of Gate pulse for Switch.

The Gate pulse of switches with phase delay is shown in Fig. 23. The switches are operated with a duty ratio of 25% and they are turned ON and OFF (altered for 50,000 times in a second) i.e., switching frequency is 50,000 Hz.

**Figure 24:**
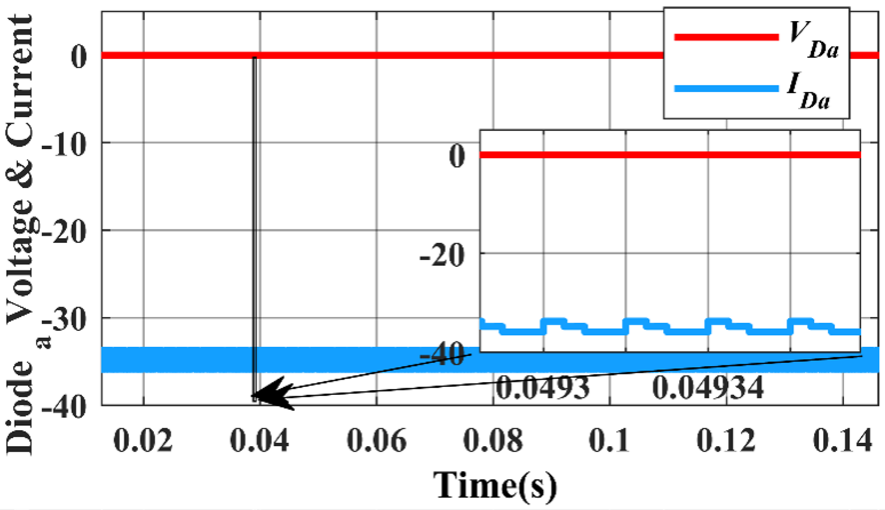
Simulation waveform of Diode a voltage and current.

The voltage and current across the diodes are shown in Fig. 24. The diodes are operated with a duty ratio of 25% and they are turned ON and OFF (altered for 50,000 times in a second) i.e., switching frequency is 50,000 Hz.

In Fig. 24, the negative voltage across diode (${d}_{a}$) is attributed to three specific cases. This occurs when the current through the diode is zero, indicating that the diode is in a reverse bias state.

**Figure 25:**
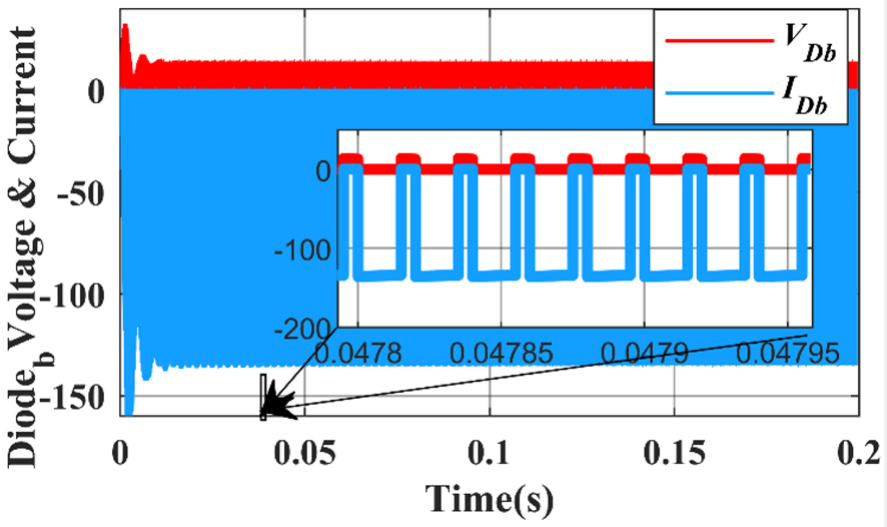
Simulation waveform of Diode b voltages and current.

In Fig. 25, The diode is initially reverse-biased, and the current is zero. At around 0.1 s, the diode becomes forward-biased, and the current begins to flow. The current increases rapidly to a peak value of 0.05 A at around 0.15 s.

**Figure 26:**
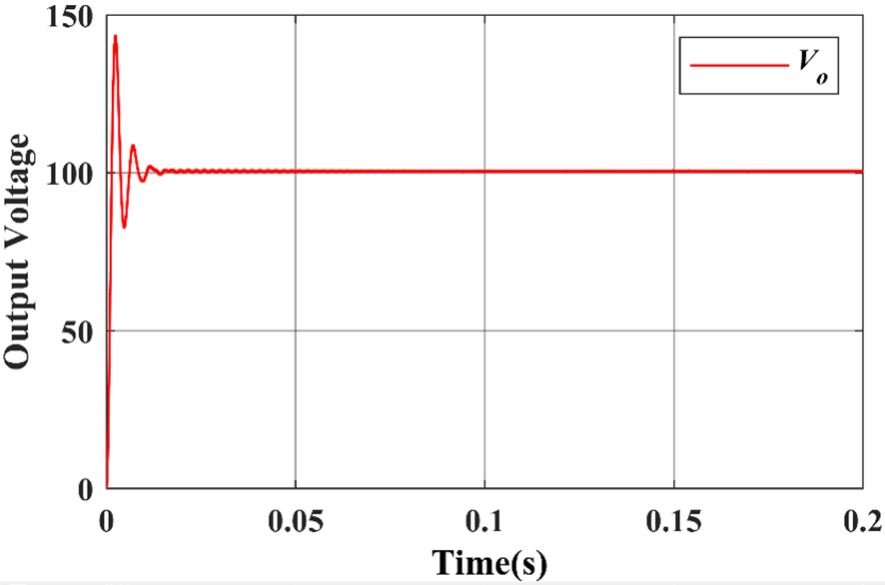
DC output voltage waveform of proposed topology.

**Figure 27:**
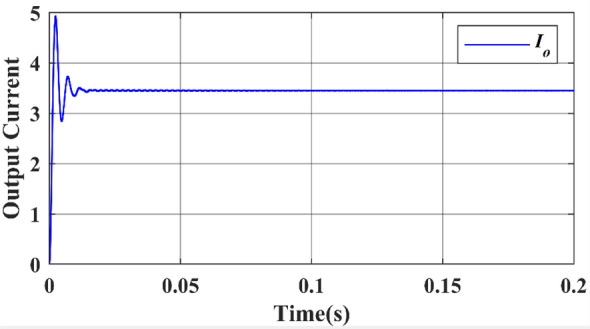
DC output current waveform of proposed topology.

Finally, the simulated output waveforms are shown in the Figs. 26 & 27 of the proposed converter. For a 100 W power, the proposed converter is designed with an output voltage of 108 V. A load of 29.1 Ω resistance is used at the output and hence the DC output current can be given as 3.468 A theoretically. Figures 26 & 27 shows the simulated output voltage waveform and DC current voltage waveform. The simulated value is approximately 100.02 V and is much closed to the computed theoretical value.

Figures 26 & 27 show that the input and output voltage waveforms of the proposed converter are related. At a frequency of 50 Hz and a duty cycle of 0.25, the output voltage consistently reaches 100 V and 3.58A.

### Performance of proposed converter

An analysis is conducted on the suggested converter's efficiency, ripple factor, output power, output current, output voltage, and the voltage stresses placed on the active and passive parts. The simulated values for various duty ratios for various circumstances are shown in Tables 7, 8 and 9.

**Table 7:** Simulated parameters for various duty ratios for case-1.

Duty cycle	${V}_{in}$	${I}_{in}$	${P}_{in}$	${V}_{0}$	${I}_{0}$	${P}_{0}$	Efficiency (%)	Ripple factor
0.1	12	3.456	41.47	33.94	1.164	39.49	95.23	1.03
0.15	12	3.455	41.46	33.93	1.163	39.47	95.21	1.03
0.2	12	3.453	41.44	33.92	1.163	39.45	95.21	0.94
0.25	12	3.453	41.44	33.91	1.163	39.44	95.18	0.91
0.3	12	3.453	41.44	33.92	1.163	39.45	95.19	0.91
0.35	12	3.453	41.44	33.92	1.163	39.45	95.19	0.91
0.4	12	3.444	41.33	33.83	1.16	39.25	94.47	0.88
0.45	12	3.444	41.33	33.83	1.16	39.25	94.97	0.73
0.5	12	3.444	41.33	33.83	1.16	39.25	94.97	0.91
0.55	12	3.45	41.41	33.89	1.162	39.39	95.12	0.83
0.6	12	3.444	41.33	33.83	1.16	39.25	94.97	0.78
0.65	12	3.444	41.33	33.83	1.16	39.25	94.97	0.81
0.7	12	3.444	41.33	33.83	1.16	39.25	94.97	0.94
0.75	12	3.444	41.33	33.83	1.16	39.25	94.97	1.06

**Table 8:** Simulated parameters for various duty ratios for case-2.

Duty cycle	${V}_{in}$	${I}_{in}$	${P}_{in}$	${V}_{0}$	${I}_{0}$	${P}_{0}$	Efficiency (%)	Ripple factor
0.1	24	6.992	167.8	68.65	2.354	161.6	96.3	1.02
0.15	24	6.989	167.7	68.62	2.343	161.5	96.28	1.02
0.2	24	6.987	167.7	68.6	2.353	161.4	96.25	1.02
0.25	24	6.986	167.7	68.6	2.352	161.4	96.25	0.8
0.3	24	6.987	167.7	68.6	2.3	161.4	96.25	0.7
0.35	24	6.987	167.7	68.6	2.353	161.4	96.25	0.8
0.4	24	6.968	167.2	68.43	2.347	160.6	96.03	0.6
0.45	24	6.968	167.2	68.43	2.336	160.6	96.04	0.6
0.5	24	6.968	167.2	68.43	2.347	160.6	96.04	0.6
0.55	24	6.981	167.5	68.55	2.351	161.1	96.18	0.6
0.6	24	6.968	167.2	68.43	2.347	160.6	96.04	0.6
0.65	24	6.968	167.2	68.43	2.347	160.6	96.04	0.6
0.7	24	6.968	167.2	68.43	2.347	160.6	96.04	0.6
0.75	24	6.968	167.2	68.43	2.347	160.6	96.04	0.6

**Table 9:** Simulated parameters for various duty ratios for case-3.

Duty cycle	${V}_{in}$	${I}_{in}$	${P}_{in}$	${V}_{0}$	${I}_{0}$	${P}_{0}$	Efficiency (%)	Ripple factor
0.1	36	10.53	379	103.4	3.544	366.3	96.66	0.967
0.15	36	10.52	378.9	103.3	3.543	366.1	96.63	0.890
0.2	36	10.52	378.7	103.3	3.543	365.9	96.61	0.871
0.25	36	10.52	378.7	103.3	3.543	365.8	96.6	0.861
0.3	36	10.52	378.7	103.3	3.543	365.9	96.61	0.919
0.35	36	10.52	378.7	103.3	3.542	365.9	96.61	0.939
0.4	36	10.49	377.7	103	3.533	364.1	96.39	0.920
0.45	36	10.49	377.7	103	3.533	364.1	96.39	0.902
0.5	36	10.49	377.7	103	3.534	364.1	96.39	0.873
0.55	36	10.51	378.4	103.2	3.539	365.3	96.54	0.968
0.6	36	10.49	377.7	103	3.533	364.1	96.4	0.873
0.65	36	10.49	377.7	103	3.534	364.1	96.4	0.970
0.7	36	10.49	377.7	103	3.534	364.1	96.4	0.951
0.75	36	10.49	377.7	103	3.534	364.1	96.4	0.922

From Table 7, the analysis of the Multi-Input SEPIC converter's performance at various duty cycles sheds light on its operation. At lower duty cycles, such as 0.1, it attains peak efficiency of 95.23% and demonstrates a low ripple factor, indicating optimal performance. However, as the duty cycle increases beyond 0.4, both efficiency and ripple factor deteriorate significantly. Input and output currents remain relatively stable across different duty cycles, while the input voltage remains constant at 12 V. This analysis underscores the critical importance of carefully selecting the duty cycle to fine-tune the Multi-Input SEPIC converter's efficiency and ripple characteristics for specific applications.

From Table 8, the analysis of the Multi-Input SEPIC converter's performance at various duty cycles sheds light on its operation. At lower duty cycles, such as 0.1, it attains peak efficiency of 96.3% and demonstrates a low ripple factor, indicating optimal performance. However, as the duty cycle decreases beyond 0.4, both efficiency and ripple factor deteriorate significantly. Input and output currents remain relatively stable across different duty cycles, while the input voltage remains constant at 24 V. This analysis underscores the critical importance of carefully selecting the duty cycle to finetune the Multi-Input SEPIC converter's efficiency and ripple characteristics for specific applications.

From Table 9, the analysis of the Multi-Input SEPIC converter's performance at various duty cycles sheds light on its operation. At lower duty cycles, such as 0.1, it attains peak efficiency of 96.66% and demonstrates a low ripple factor, indicating optimal performance. However, as the duty cycle increases beyond 0.4, both efficiency and ripple factor deteriorate significantly. Input and output currents remain relatively stable across different duty cycles, while the input voltage remains constant at 36 V. This analysis underscores the critical importance of carefully selecting the duty cycle to fine-tune the Multi-Input SEPIC converter's efficiency and ripple characteristics for specific applications.

It can be observed that the highest efficiency point is achieved at duty ratio 0.1 < d > 0.75. The proposed converter is designed with duty ratio of 25% but the highest efficiency point might occur at this duty ratio. It is also observed that the efficiency is quite higher (96%) even at lower duty ratios i.e., from 10 to 75%. And the ripple factor is under the universal limit point that is below 10% percent up to the duty ratio of 75%.

**Figure 28:**
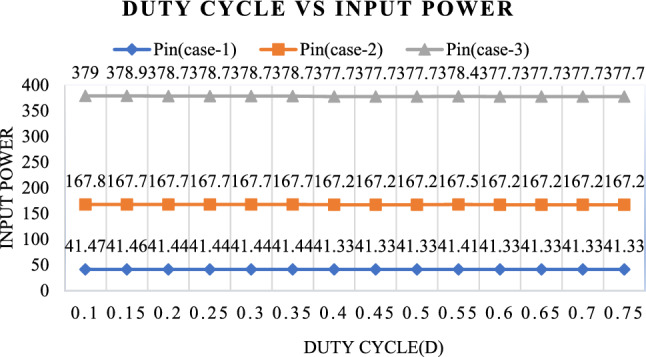
Relationship between input power and different duty ratios.

Figure 28 shows the Input power for three cases with different duty cycles. It is observed that the Input power remains constant for various duty cycles, ranging from 0.1 to 0.75, in all three cases.

**Figure 29:**
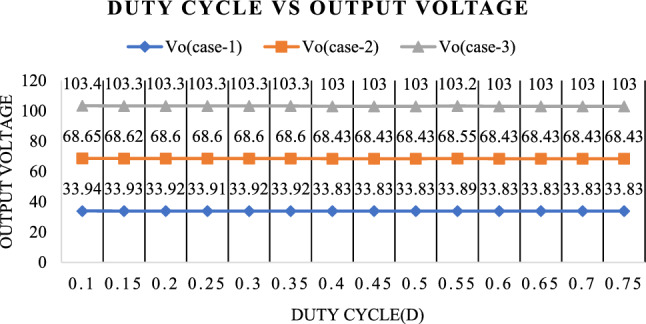
Relationship between output voltage and different duty ratios.

The output voltage for three scenarios with various duty cycles is displayed in Fig. 29. In all three scenarios, it is seen that the output voltage stays constant across a range of duty cycles, from 0.1 to 0.75.

**Figure 30:**
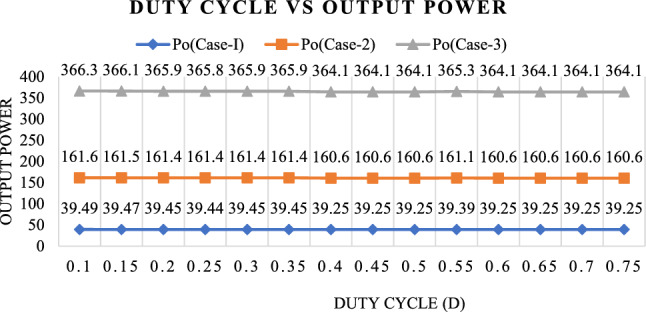
Relationship between output power and different duty ratios.

Figure 30 shows the Output power for three cases with different duty cycles. It is observed that the Output power remains constant for various duty cycles, ranging from 0.1 to 0.75, in all three cases.

**Figure 31:**
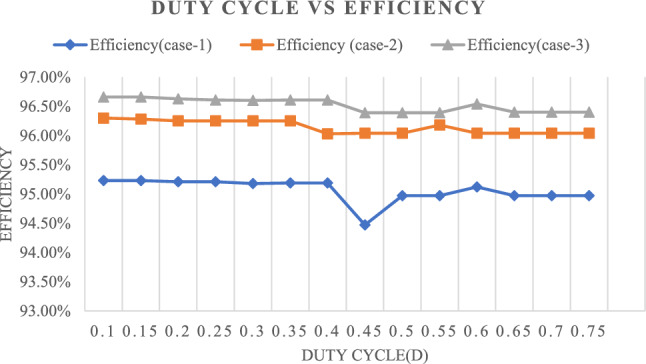
Relationship between efficiency and different duty ratios.

Figure 31 shows the efficiency for three cases with different duty cycles. It is observed that the efficiency remains constant for various duty cycles, ranging from 0.1 to 0.75, in all three cases.

**Figure 32:**
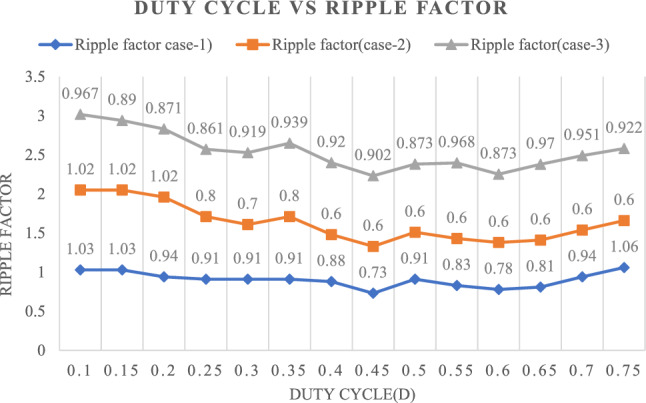
Relationship between ripple factor and different duty ratios.

Figure 32 shows the Ripple factor for three cases with different duty cycles. It is observed that the Ripple factor remains constant for various duty cycles, ranging from 0.1 to 0.75, in all three cases.

### Comparisons with existing topologies

The Table 10 initially shows that the proposed converter can be compared with various traditional converters, offering significant advantages and finding numerous applications^31^. This proposed converter features an equal number of sources, high efficiency, and fewer diodes and relays. It also exhibits lower voltage stress than other converters^34^. In terms of the number of power switches (bidirectional), it stands out by requiring only two switches, while other converters typically have more^36^. The Multi-Input SEPIC converter boasts fewer diodes compared to traditional converters, which often require 5 or 3 diodes^33^. Furthermore, the Multi-Input SEPIC converter achieves an efficiency of over 96%, surpassing the lower efficiency percentages of 95%, 94%, and 88%-94% seen in other converters^35^. When comparing these aspects with the Multi-Input SEPIC converter, it becomes evident that it excels in numerous aspects and applications^34^.

**Table 10:** Evaluation of the suggested converter in comparison to other topologies.

Topology	Relation between input and output voltages	No of sources	No of power switches (unidirectional)	No of power switches (bidirectional)	No of relays	No of diodes	% efficiency	Voltage stress	No of capacitors	No of inductors
^31^	${V}_{0}= \frac{\left( {d}_{1}+ {d}_{2}\right){V}_{1}+{d}_{2}{V}_{2}}{1-{d}_{1}-{d}_{2}}$	2	0	3	0	5	93.50	High	2	2
^32^	${V}_{0}= \frac{{V}_{1}{d}_{1}+\left( {V}_{1}+ {V}_{2}\right){d}_{2}+ {V}_{2}{d}_{3}}{1-{d}_{1}-{d}_{2}-{d}_{3}}$	2	4	0	4	3	94	–	1	1
^33^	${V}_{0}= \frac{{d}_{1}{V}_{1}+(1- {d}_{1 }){V}_{2}}{1- {d}_{2}}$	2	0	3	3	1	93	–	1	1
^34^	${V}_{0}= - \frac{{V}_{1}{d}_{1}+\left( {V}_{1}+ {V}_{2}\right){d}_{3}+ {V}_{2}{d}_{2}}{1-{d}_{1}-{d}_{2}-{d}_{3}}$	2	2	2	0	0	94	High	1	1
^35^	During discharging ${V}_{0}= \frac{{V}_{battery }{d}_{1}+\left(1-{d}_{1}\right) {V}_{1}+(1-{d}_{2}){V}_{2}}{1-{d}_{3}}$ During charging ${V}_{0}= \frac{-{V}_{battery }{d}_{1}+ {V}_{1}+(1-{d}_{2}){V}_{2}}{1-{d}_{3}}$	3	4	2	0	2	88–94	Low	1	1
^36^	${V}_{0}= \frac{\left(2-{d}_{1}\right){V}_{1}+{V}_{2}}{(1-{d}_{1}{)}^{2}}$	2	6	2	0	2	91	High	4	4
^37^	${V}_{0}= \frac{\left(2-{d}_{1}\right){V}_{1}+{V}_{2}}{(1-{d}_{1}{)}^{2}}$	2	4	0	0	4	95	Moderate	4	4
^38^	${V}_{0}= \frac{\left(2-{d}_{1}\right){V}_{1}+{V}_{2}}{(1-{d}_{1}{)}^{2}}$	2	3	0	0	3	94	Moderate	3	3
proposed	${V}_{0}= \frac{{d}_{1}+ {d}_{2}+ {d}_{3}}{1-{d}_{1}-{d}_{2}-{d}_{3}} \times$ (${V}_{1}\left({d}_{1}+ {d}_{3}\right)+ {V}_{2}\left({d}_{2}+ {d}_{3}\right))$	2	4	0	0	2	96	Low	2	2

**Figure 33:**
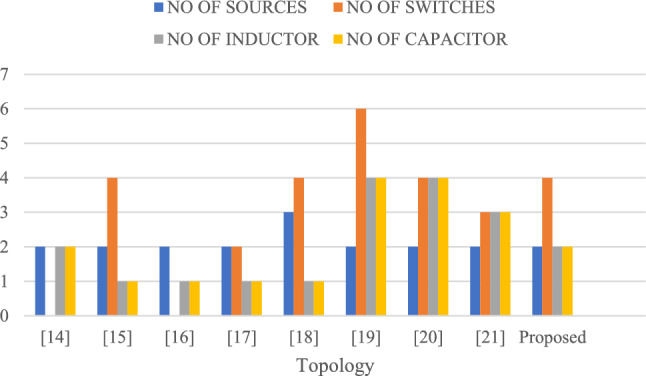
Comparison between no of sources, no of switches, no of inductors, no of capacitors for different topology.

**Figure 34:**
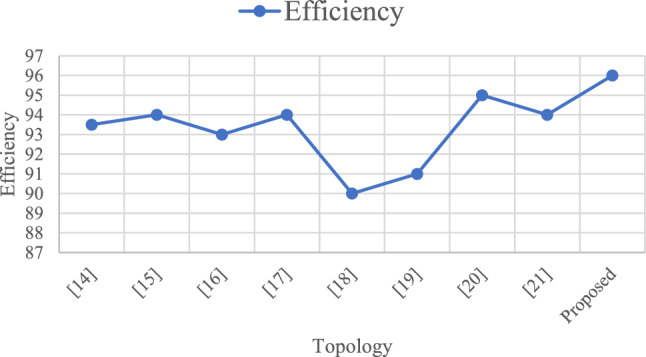
Comparison between efficiency for different topology.

**Figure 35:**
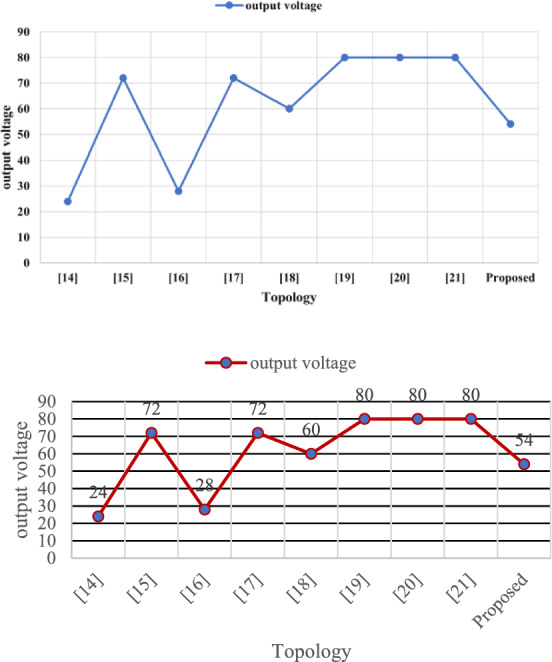
Comparison between output voltage for different topology.

Figures 33, 34, and 35 present a comprehensive comparison of various converters with the proposed Multi-Input SEPIC converter across different aspects. The data clearly demonstrates that the Multi-Input SEPIC converter out performs all traditional converters.

## Conclusion

In conclusion, our study presents a highly efficient SEPIC converter integrated with a multi-input DC–DC configuration for DC microgrid management. Using Simulink in MATLAB, we verified its ability to provide continuous power to the load by utilizing both a DC source 1 and DC source 2. The configuration versatility allows operation across a wide range of output voltages while maintaining effective power regulation. Various modelling techniques were employed to ensure precise design and analysis, including Average large-signal, small-signal, and steady-state modelling. Stability was assessed using the R–H stability criterion, and the output voltage expression was derived from steady-state modelling. Our study discusses the converter's operation and the role of switches in power transmission from single or dual sources. Efficiency comparisons with established converter topologies demonstrated an impressive 95% efficiency at rated load, with minimal system losses. The Multi-Input SEPIC converter's effectiveness, design, performance, and stability insights position it as a promising solution for efficient DC power microgrid management. Its potential for sustainable and reliable power supply solutions becomes particularly evident when integrated with renewable energy sources.

## Data Availability

The datasets used and/or analyzed during the current study available from the corresponding author on reasonable request.

## References

[1] Duan Y, Zhao Y, Hu J (2023). An initialization-free distributed algorithm for dynamic economic dispatch problems in microgrid: Modeling, optimization and analysis. Sustain. Energy Grids Netw..

[2] Shen Y, Liu D, Liang W, Zhang X (2023). Current reconstruction of three-phase voltage source inverters considering current ripple. IEEE Trans. Transp. Electr..

[3] Basha CHH, Rani C, Odofin S (2017). A review on non-isolated inductor coupled DC–DC converter for photovoltaic grid-connected applications. Int. J. Renew. Energy Res. (IJRER).

[4] Reddy KR (2023). A novel on energy management strategy with maximum exploitation of renewables and EV storage in distribution networks. Int. Trans. Electr. Energy Syst..

[5] Prashanth V (2024). Implementation of high step-up power converter for fuel cell application with hybrid MPPT controller. Sci. Rep..

[6] Basha CHH, Rani C (2022). A new single switch DC–DC converter for PEM fuel cell-based electric vehicle system with an improved beta-fuzzy logic MPPT controller. Soft Comput..

[7] Gao Y, Doppelbauer M, Ou J, Qu R (2021). Design of a double-side flux modulation permanent magnet machine for servo application. IEEE J. Emerg. Sel. Top. Power Electron..

[8] Kiran, S. R. *et al*. Design and performance analysis of hybrid optimization MPPT controller for proton exchange membrane fuel cell system with DC–DC converter. *Mater. Today Proc*. (2023).

[9] Li P, Hu J, Qiu L, Zhao Y, Ghosh BK (2022). A distributed economic dispatch strategy for power-water networks. IEEE Trans. Control Netw. Syst..

[10] Shirkhani M, Tavoosi J, Danyali S, Sarvenoee AK, Abdali A, Mohammadzadeh A, Zhang C (2023). A review on microgrid decentralized energy/voltage control structures and methods. Energy Rep..

[11] Velpula, S. *et al*. Impact of DFIM controller parameters on SSR characteristics of wind energy conversion system with series capacitor compensation. in *International Conference on Computer Vision and Robotics*. (Springer, 2023).

[12] Puppala R (2023). Framework for smart grid to implement a price elasticity-based peak time rebate demand response program. Front. Energy Res..

[13] Hou M, Zhao Y, Ge X (2017). Optimal scheduling of the plug-in electric vehicles aggregator energy and regulation services based on grid to vehicle. Int. Trans. Electr. Energy Syst..

[14] Rafikiran S (2023). Design and implementation of hybrid MPPT controller for FC based EV system with boost DC–DC converter. J. Intell. Fuzzy Syst..

[15] Zhou S, Zhou G, Liu X, Zhao H (2023). Dynamic freewheeling control for SIDO buck converter with fast transient performance, minimized cross-regulation, and high efficiency. IEEE Trans. Ind. Electron..

[16] Chen J, Xu J, Zhang Y, Zhao J, Hou J, Wang Y (2024). Geometrical state-plane-based synchronous rectification scheme for LLC converter in EVs. IEEE Trans. Transp. Electr..

[17] Kumari PA (2024). Application of DSO algorithm for estimating the parameters of triple diode model-based solar PV system. Sci. Rep..

[18] Hussaian Basha CH, Rani C (2020). Performance analysis of MPPT techniques for dynamic irradiation condition of solar PV. Int. J. Fuzzy Syst..

[19] Najafzadeh M, Ahmadiahangar R, Husev O, Roasto I, Jalakas T, Blinov A (2021). Recent contributions, future prospects and limitations of interlinking converter control in hybrid AC/DC microgrids. IEEE Access.

[20] Basha CH, Rani C (2020). Design and analysis of transformerless, high step-up, boost DC–DC converter with an improved VSS-RBFA based MPPT controller. Int. Trans. Electr. Energy Syst..

[21] Gu W, Zhang D (2008). Designing a SEPIC converter. Excell. Des. Guidel..

[22] Li X, Yang J, Chen J (2022). A hybrid DC–DC converter based on buck and boost converters for DC microgrids with multiple DC sources and energy storage systems. Renew. Energy.

[23] Durán E, Litrán SP, Ferrera MB (2020). An interleaved single-input multiple-output DC–DC converter combination. CSEE J. Power Energy Syst..

[24] Basha CH, Rani C (2020). Different conventional and soft computing MPPT techniques for solar PV systems with high step-up boost converters: A comprehensive analysis. Energies.

[25] Seguel JL, Seleme SI, Morais LMF (2022). Comparative study of buck-boost, SEPIC, cuk and zeta DC–DC converters using different MPPT methods for photovoltaic applications. Energies.

[26] Zakaria A, Marei MI, Mashaly HM (2023). A hybrid interleaved DC–DC converter based on buck-boost topologies for medium voltage applications. E-Prime.

[27] Mahafzah KA, Obeidat MA, Al-Shetwi AQ, Ustun TS (2022). A Novel synchronized multiple output DC–DC converter based on hybrid flyback-cuk topologies. Batteries..

[28] Dahono A, Rizqiawan A, Dahono PA (2020). A modified Cuk DC–DC converter for DC microgrid systems. TELKOMNIKA Telecommun. Comput. Electron. Control.

[29] İskender İ, Genç N (2019). Power electronic converters in DC microgrid. Power Syst..

[30] Dahale, S., Das, A., Pindoriya, N. M. & Saravanakumar, R. An overview of DC–DC converter topologies and controls in DC microgrid. in *2017 7th International Conference on Power Systems (ICPS)* (2017).

[31] Vinothkumar A, Deepika MS, Priya VK, Jayasri VG (2023). A PFC based EV battery charger using a bridgeless isolated SEPIC converter. Int. J. Res. Appl. Sci. Eng. Technol..

[32] Kumaravel, S., Kumar, G. G., Veeranna, K. & Karthikeyan, V. Novel non-isolated modified interleaved DC–DC converter to integrate ultracapacitor and battery sources for electric vehicle application. In *2018 20th National Power Systems Conference (NPSC)*, 1–6 (2018).

[33] Divya G, Sridharan S, Velmurugan P (2023). Integrated interleaved luo converter with buck converter designed for electric vehicle application. IET Power Electron..

[34] Athikkal S, Kumar GG, Kumaravel S, Ashok S (2019). A non-isolated bridge-type DC–DC converter for hybrid energy source integration. IEEE Trans. Ind. Appl..

[35] Varesi K, Hosseini SH, Sabahi M, Babaei E (2017). Performance analysis and calculation of critical inductance and output voltage ripple of a simple non-isolated multi-input bidirectional DC–DC converter. Int. J. Circuit Theory Appl..

[36] Hussaian Basha, C. H. *et al*. Design of high voltage gain DC–DC converter with fuzzy logic controller for solar PV System under dynamic irradiation conditions. in *Proceedings of the International Conference on Paradigms of Computing, Communication and Data Sciences: PCCDS 2022*. (Springer, 2023).

[37] De Alcantara Bastos GH, Costa LF, Tofoli FL, Bascope GVT, Bascopé RPT (2020). Nonisolated DC–DC converters with wide conversion range for high-power applications. IEEE J. Emerg. Sel. Top. Power Electron..

[38] Mariprasath T (2024). A novel on high voltage gain boost converter with cuckoo search optimization based MPPT controller for solar PV system. Sci. Rep..

[39] Touti E (2024). A novel design and analysis adaptive hybrid ANFIS MPPT controller for PEMFC-Fed EV systems. Int. Trans. Electr. Energy Syst..

[40] Basha, C. H., Rani, C. & Odofin, S. Analysis and comparison of SEPIC, Landsman and Zeta converters for PV fed induction motor drive applications. in *Proceedings of the 2018 International Conference on Computation of Power, Energy, Information and Communication (ICCPEIC)* (2018).

[41] Bairabathina S, Balamurugan B (2022). Design and validation of a SEPIC-based novel multi-input DC–DC converter for grid-independent hybrid electric vehicles. Energies.

[42] Jabbar A, Mansor M, Jaafar S (2018). Single-ended primary inductor converter (SEPIC) for LED application. IET Circuits Dev. Syst..

[43] Narendranath KV, Viswanath Y, Babu KS, Arunkumar G, Elangovan D (2017). Solar fed DC–DC single ended primary inductance converter for low power applications. IOP Conf. Ser. Mater. Sci. Eng..

[44] Kiran SR (2022). Reduced simulative performance analysis of variable step size ANN based MPPT techniques for partially shaded solar PV systems. IEEE Access.

[45] Bhattacharjee AK, Kutkut NH, Batarseh I (2019). Review of multiport converters for solar and energy storage integration. IEEE Trans. Power Electron..

